# hESCs‐derived Organoids Achieve Liver Zonation Features through LSEC Modulation

**DOI:** 10.1002/advs.202411667

**Published:** 2025-04-25

**Authors:** Yuying Zhang, Chenyan Huang, Lei Sun, Lyu Zhou, Yudi Niu, Kaini Liang, Bingjie Wu, Peng Zhao, Zhiqiang Liu, Xiaolin Zhou, Peng Zhang, Jianchen Wu, Jie Na, Yanan Du

**Affiliations:** ^1^ School of Biomedical Engineering, Tsinghua‐Peking Center for Life Sciences Tsinghua University Beijing 100084 China; ^2^ National Key Laboratory of Kidney Diseases Beijing China; ^3^ School of Basic Medical Science, Tsinghua Medicine Tsinghua University Beijing 100084 China; ^4^ Department of Molecular Biology Princeton University Princeton NJ 08544 USA; ^5^ Institution of Medical Science University of Toronto Toronto Ontario M5S1A8 Canada; ^6^ Beijing Key Laboratory for Genetics of Birth Defects, Beijing Pediatric Research Institute, MOE Key Laboratory of Major Diseases in Children; Rare Disease Center, Beijing Children's Hospital, Capital Medical University, National Center for Children's Health Beijing 100045 China

**Keywords:** GLP‐1R, hESCs‐derived LSEC, liver zonation, MAFLD, PEGMA‐grafted PDMS microwell chip, zonated organoids

## Abstract

Liver zonation, essential for diverse physiological functions, is lacking in existing organoid models, hindering their ability to recapitulate liver development and pathogenesis. Addressing this gap, this work explores the feasibility of achieving zonated organoid by co‐culturing human embryonic stem cells (hESCs) derived hepatocytes (HEP) with hESCs derived liver sinusoidal endothelial cells (LSECs) exhibiting characteristics of either the liver lobule's pericentral (PC) or periportal (PP) regions. Introducing zonated LSECs with variable WNT2 signaling subtly regulate hepatocyte zonation, resulting in noticeable metabolic function changes. Considering the lipid metabolism variations in PC and PP organoids, this work constructs biomimetic zonated metabolic dysfunction‐associated steatotic liver disease (MASLD) organoids and revealed that glucagon‐like peptide‐1 receptor agonist (GLP‐1RA) directly target LSECs, indicating potential therapeutic mechanisms of GLP‐1RA in MAFLD alleviation. This study highlights the crucial role of non‐parenchymal cells in organoids for recapitulating niche heterogeneity, offering further insights for drug discovery and in vitro modeling of organ heterogeneity.

## Graphical abstract

This article addresses the absence of zonation features in current organoid models by co‐culturing human embryonic stem cell‐derived hepatocytes with liver sinusoidal endothelial cells exhibiting distinct zonal characteristics. It demonstrates that differential WNT2 signaling from endothelial cells modulates hepatocyte zonation and metabolic functions, facilitating the construction of biomimetic MAFLD models and providing mechanistic insights into GLP‐1RA therapies.

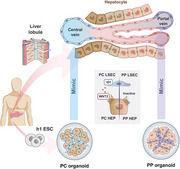

## 1 Introduction

The unique architecture of liver, comprised of hundreds of thousands of basic units called hepatic lobules, enables it to perform numerous vital physiological functions essential for body homeostasis. The lobule is divided into periportal and pericentral areas, with mid‐lobule cells in between, exhibiting spatial diversity known as zonation, characterized by variations in gene expression profiles and functional attributes among hepatocytes and non‐parenchymal cells. Such non‐uniform distribution of biological activities optimizes the general liver function and enables flexible response to the change of body requirements. Many liver diseases also showed zonated pathologies. For example, liver parenchymal damage resulted from drug overdose and MASLD usually initiate from pericentral regions,^[^ ^1^, ^2^ ^]^ while autoimmune hepatitis and primary bile cirrhosis preferentially initiate from periportal regions.^[^ ^3^, ^4^ ^]^

The current approach of studying the liver as a uniform entity fails to capture its intricate complexity, resulting in the loss of vital information derived from spatial heterogeneity. Even though cells in organoid models present a homogenous identity and metabolic profile, it's imperative that these models are refined to better represent the heterogeneity of liver cells in various in vivo zones. Hepatocyte zonation is mainly regulated by LSEC‐derived angiocrine Wnt factors.^[^ ^5^, ^6^, ^7^ ^]^ Pericentral LSECs, as the main source of angiocrine Wnt‐pathway ligands (e.g., Wnt2, Wnt9b, and Rspo3), induce β‐catenin activation in adjacent hepatocytes, which leads to the expression of pericentral‐specific functions, such as glutamine synthesis (GS), cytochrome P450 family members Cyp2e1 (for lipid metabolism), E‐cadherin (Bile acid synthesis and transport) and others.^[^ ^7^ ^]^ Specific ablation of angiocrine Wnt signaling results in decreased β‐catenin activation and consequently perturbed liver metabolic zonation, as indicated by upregulation of periportal transcripts in pericentral hepatocytes (e.g., Arginase 1).^[^ ^8^, ^9^ ^]^ Although hepatic heterogeneity has been widely investigated, it remains obscure as how LSECs, the key regulator of liver zonation, obtain their zonated profiles via various signaling cues. A better understanding of the angiodiversity of LSECs as well as the molecular programs that drive their development and zonation may pave the way for more robust differentiation of human pluripotent stem cells (hPSCs) to LSECs and, hopefully, lead to advances in success generation of liver organoids with zonated features and precision therapies for multiple liver diseases.

It can be noted that the origin of LSECs remains incompletely understood. Studies conducted in model organisms such as zebrafish, mice, birds, as well as human fetal liver samples suggest potential origins from veins, mesothelial cells, and the endodermal layer.^[^ ^10^, ^11^, ^12^, ^13^, ^14^, ^15^ ^]^ There are relatively few successful studies demonstrating the differentiation of LSECs from hPSCs. Koui et al. reported the first differentiation of hPSCs into cells resembling LSECs.^[^ ^16^ ^]^ They initially differentiated hPSCs into mesodermal cells, further differentiating them into endothelial progenitors. Subsequently, CD34+, CD31+, and FLK1+ LSEC progenitor cells were isolated through marker selection and were induced to overexpress LSEC markers STAB2, F8, LYVE1, and CD32B by inhibiting the TGFβ signaling pathway. Gage et al. published a second protocol for differentiating functional LSECs from hPSCs.^[^ ^17^ ^]^ They found that LSECs derived from venous endothelium exhibited higher levels of marker gene expression (e.g., CD32B and STAB2) and stronger ligand‐binding capabilities. However, both studies did not compare and correlate the characteristics of hPSCs‐derived LSECs with those of in vivo LSEC subpopulations located in different zones of the liver lobules.

A more comprehensive profiling of LSEC zonation is made possible by high‐resolution spatial gene expression mapping with scRNAseq. Using paired‐cell RNA sequencing that achieves spatial inference of LSECs based on the expression of landmark genes in their adjacent hepatocytes, Halpern et al. reconstructed the zonation profiles of 475 genes that are significantly zonated in LSECs (mouse) and did further validation of 12 zonated genes using smFISH.^[^ ^18^ ^]^ Moreover, a recent study conducted by Aizarani et al. provided the single‐cell transcriptome‐derived zonation profiles of human LSECs, indicating limited evolutionary conservation of LSEC zonation pattern between human and mouse.^[^ ^19^ ^]^ In addition to well‐investigated WNT2, WNT9B and RSPO3, genes such as FCN3, ICAM1 and ENG were also suggested to be part of the potential molecular signature of liver pericentral niche.^[^ ^19^ ^]^ Meanwhile, BTNL9 and ANPEP are thought to be potential molecular features around the periportal niche.^[^ ^19^ ^]^

Acknowledging that in vivo compartmentalized LSECs might be one of the key regulators of hepatocyte zonation, we first generated hESCs derived PP and PC LSECs and subsequently explore the feasibility of creating 3D zonated liver organoids. 3D organoid culture system that facilitates the uniform aggregation of LSECs and hepatocytes was constructed by utilizing poly (ethylene glycol) methyl ether acrylate (PEGMA) grafted polydimethylsiloxane (PDMS) microwell chip. By employing immune‐fluorescence imaging and metabolic function characterization, we confirmed that co‐culturing zonated LSECs with differential WNT2 signaling resulted in changes in hepatocyte zonation profiles. In an application involving zonated organoids, it was notably observed that GLP‐1RA could alleviate MASLD features by targeting GLP‐1R expressed on zonated PC and PP LSECs, but not on hepatocytes. This finding provides a potential explanation for the therapeutic mechanism of GLP‐1RA in the treatment of MASLD.

## 2 Results

### 2.1 Developing Efficient Culture System for hESCs‐Derived Zonated LSECs

**Figure 1:**
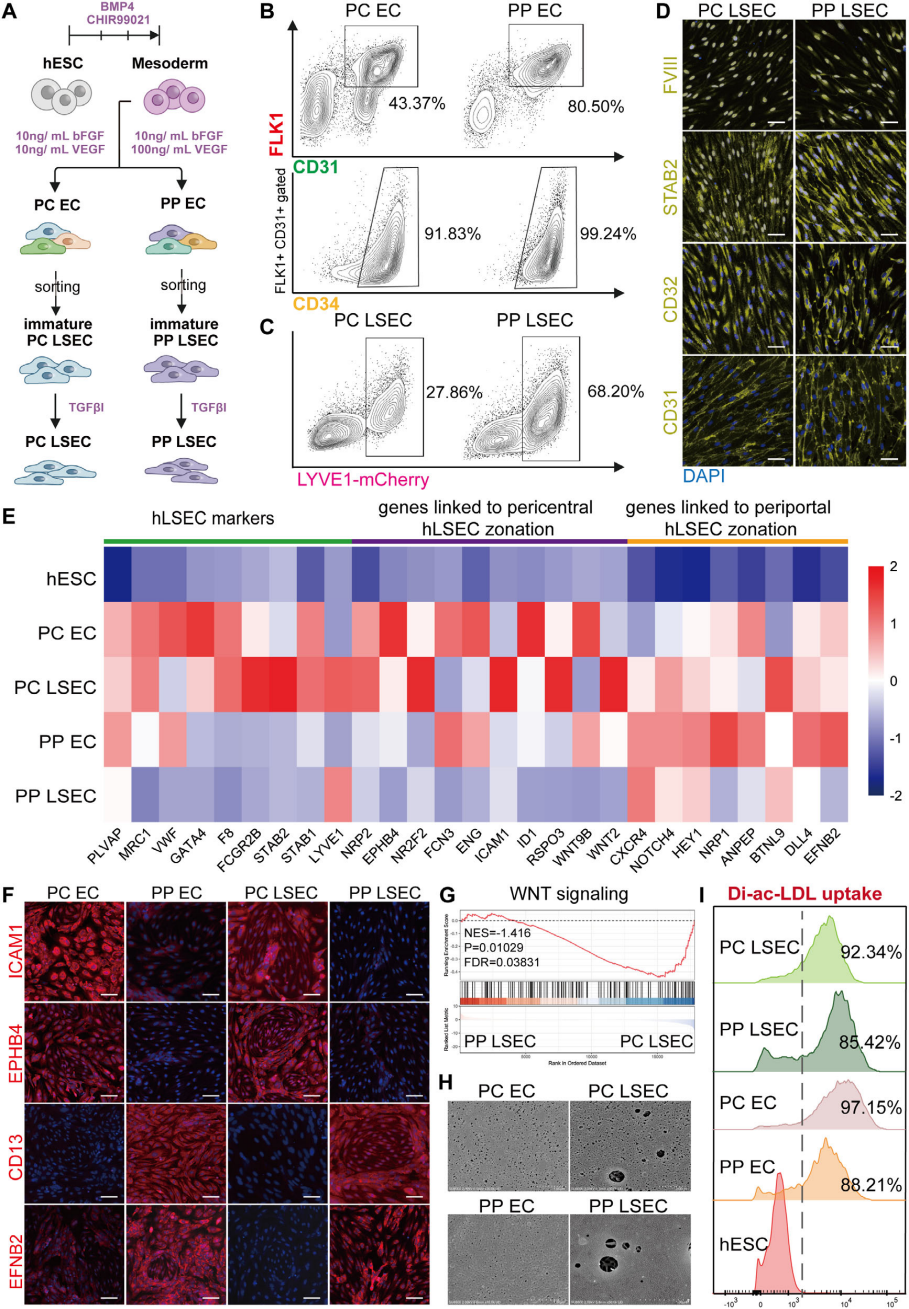
Development of efficient cell culture system for hESCs‐derived zonated LSECs. A) The protocol illustration of inducing PC and PP LSECs from hESCs. B) The proportion of FLK1+CD31+CD34+ immature LSECs within PC and PP EC populations. C) The proportion of LYVE1‐mCherry+ LSECs within mature PC and PP LSEC populations. D) Immunofluorescence staining of general EC marker (CD31) and three human LSEC markers (STAB2, CD32, FVIII) for hESC‐derived PC/PP LSECs, with scale bar of 50 µm. E) RNA sequencing analyzed the expression of human LSEC marker genes and LSEC zonation related genes for hESC‐derived PC/PP ECs and PC/PP LSECs, and the heatmap displays the average results from three replicate experiments. F) Immunofluorescence staining of human primary PC LSEC high expressed proteins (ICAM1, EPHB4) and primary PP LSEC high expressed proteins (CD13, EFNB2) for hESC‐derived PC/PP ECs and PC/PP LSECs, with scale bar of 50 µm. G) Gene set enrichment analysis of WNT signaling pathway in hESC‐derived PC LSEC and PP LSEC. H) SEM observation for the fenestration of hESC‐derived PC/PP ECs and PC/PP LSECs. I) FACS quantitative analysis of the proportion of cells phagocytosing DiI‐Ac‐LDL in hESC‐derived PC/PP ECs and PC/PP LSECs.

The optimization of the method for differentiating LSECs from hESCs was based on previously published work.^[^ ^16^, ^17^, ^20^, ^21^ ^]^ The basal culture medium used was a cost‐effective, chemically defined homemade AATS medium, which consisted of RPMI 1640 supplemented with rice‐derived recombinant human albumin, l‐ascorbic acid 2‐phosphate magnesium salt, human Apo‐transferrin, and sodium selenite.^[^ ^21^ ^]^ Analysis of publicly available single‐cell sequencing data^[^ ^19^ ^]^ revealed that primary human PC LSECs exhibit a high expression of WNT signaling and a gene expression profile biased toward venous endothelial identity (Figure S1, Supporting Information), whereas primary human PP LSECs show a low expression of WNT signaling and a gene expression profile biased toward arterial endothelial identity.^[^ ^22^ ^]^ Following the methodology reported by Gage et al.,^[^ ^17^ ^]^ we differentiated LSECs from hESC derived arterial and venous angioblasts separately (**Figure** **1**A). The differentiation process was divided into three sequential stages: (a) hESCs were differentiated into mesodermal cells by the addition of BMP4 and CHIR99021 (days 0–3); (b) Subsequently, VEGF and bFGF were added to further differentiate mesodermal cells into FLK1+CD31+CD34+ LSEC progenitor cells (days 3–6); (c) Finally, the addition of TGFβ inhibitor SB431542 facilitated the maturation of LSEC progenitor cells, resulting in the expression of markers LYVE1, CD32, STAB2, and F8 in the LSECs (days 6–12) (Figure 1A). At day 6, ≈40% of FLK1+ CD34+ CD31+ PC LSEC progenitor cells were observed in the venous‐like population, whereas the arterial‐like population contained ≈80% of FLK1+ CD34+ CD31+ PP LSEC progenitor cells (Figure 1B). To determine the maturation of progenitor cells, we established a mCherry reporter hESC line by visualizing the expression of a common marker of the LSECs, LYVE1—using CRISPR–Cas9 genome editing. At day12, the venous‐like population exhibited less than 30% differentiation of FLK1+ CD34+ CD31+ progenitor cells into LYVE1+ LSECs, whereas the efficiency in the arterial‐like population approached ≈70% (Figure 1C). Immunofluorescence staining and RNA‐seq analysis revealed that both PC LSEC and PP LSEC expressed CD31, LYVE1, CD32B, STAB2, and F8 (Figure 1D,E and Figure S2A, Supporting Information).

In PC EC and PC LSEC, venous marker genes (i.e., *NRP2*, *NR2F2*, *EPHB4*) showed significantly higher expression than in their PP counterparts, while arterial marker genes (i.e., *EFNB2*, *DLL4*, *NRP1*, *CXCR4*) exhibited lower expression levels (Figure 1E and Figure S2A, Supporting Information). We investigated features associated with LSEC zonation and observed elevated expression levels in PC LSEC for pericentral hepatocyte zonation regulators (i.e., *WNT2*, *WNT9B*, *RSPO3*), in vivo pericentral region high expression genes (i.e., *ICAM1*, *ENG*) and the majority of glycolysis‐related metabolites (Figure 1E and Figure S2B, Supporting Information). Conversely, in PP LSEC, we observed higher expression levels of in vivo periportal‐enriched genes, including *EFNB2* and *DLL4*, along with genes associated with oxidative phosphorylation pathways, compared to PC LSEC (Figure 1E and Figure S2C, Supporting Information). Moreover, immunofluorescence staining analysis also revealed significantly higher expression of EPHB4 and ICAM1 in PC EC and PC LSEC compared to their PP counterparts, while EFNB2 and CD13 exhibited lower expression levels (Figure 1F). The Pearson correlation analysis demonstrated that hESC‐derived PC LSECs closely resemble in vivo pericentral zone human LSECs and exhibit a slight positive correlation with periportal zone human LSECs, yet show no correlation with other liver cell types (Figure S1D, Supporting Information). In a similar vein, hESC‐derived PP LSECs align most closely with in vivo periportal zone human LSECs (Figure S1D, Supporting Information). Furthermore, PC LSECs and PP LSECs exhibit differential activation of WNT signaling while both retaining the typical fenestrations of in vivo liver sinusoidal endothelial cells (Figure 1G,H). Additionally, the acetylated low‐density lipoprotein (acLDL) uptake assay confirmed that both PC LSECs and PP LSECs demonstrate the capacity to phagocytose acetylated low‐density lipid (Figure 1I and Figure S2D, Supporting Information). Taken together, we validated that the PC and PP LSECs generated through VEGF pathway regulation exhibit gene expression, metabolic states, and hepatocyte zonation regulator differences similar to in vivo pericentral and periportal region LSECs.

### 2.2 3D Liver Organoids Formation within PEGMA‐Grafted PDMS Microwell Chip

**Figure 2:**
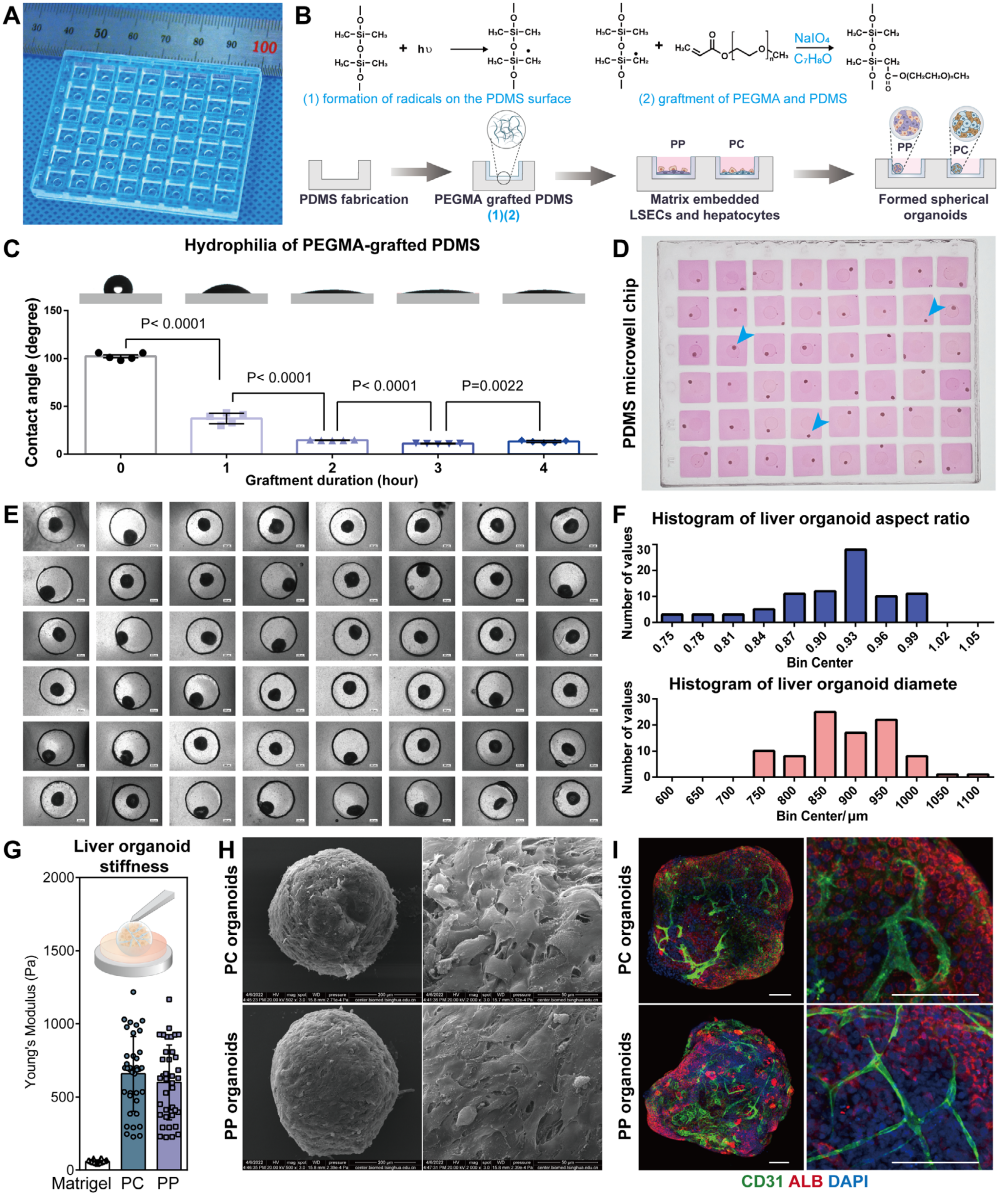
PEGMA‐grafted PDMS microwell chips for low‐attachment liver organoid culture. A) Photo of the PDMS chip. B) Schematic illustration of the operation process of the 3D culture system: fabrication of the PDMS chip; PEGMA grafting onto PDMS; assembling hESC‐derived PC/PP LSECs and hepatocytes with collagen I and Matrigel; formation of PC/PP organoids. C) Contact angle measurement of PDMS surfaces with varying grafting times, from 0 to 4 h. Measurements were conducted in accordance with ASTM D5946‐17, using a 2 µL water droplet. Significance was determined using one‐way ANOVA, selecting for comparison between adjacent groups (*n* = 5). D) Image of 3D organoids cultured on a PEGMA‐grafted PDMS microwell chip, and the blue arrows indicate the organoids. E) Enlarged bright‐field images showing organoid formation 48 h after cell seeding, with a 500 µm scale bar. F) Histogram analysis of spheroid dimensions, which presents the distribution of aspect ratios and diameters for a total of ninety‐two spheroids. The x‐axis represents the measured aspect ratios and diameters, while the y‐axis counts the number of spheroids falling within each specified range. G) Determination of 3D PC and PP organoid stiffness by AFM. The Young's modulus was measured randomly at various locations within each organoid, with twenty measurements per location. The average measurements from three replicate experiments was recorded as the displayed result (*n* = 30). H) SEM observation for the cell and ECM distribution of PC and PP organoids. I) 3D visualization of immunofluorescence staining for CD31 (indicating vascular structure) and ALB (indicating hepatocyte function) in PC and PP organoids, with a scale bar of 200 µm.

To engineer and culture the liver organoids formed by hESC‐derived zonated LSECs and hepatocytes, we created a low‐attachment micro‐well chip (Figures S3 and S4, Supporting Information). The micro‐well chip is comprised of two components: an array of two‐layer wells made from PDMS (**Figure** **2**A), which serves to confine the geometric shape of the introduced cells and matrix, and PEGMA modification on the surface of PDMS, enabling low adhesion and promoting the formation of spherical organoids (Figure 2B). The process of PEGMA graftment involves first generating radicals on the PDMS surface under UV light exposure, followed by the polymerization reaction between PEGMA monomers and PDMS (Figure 2B). From the contact angle measurements, it was evident that the surface hydrophilicity of PDMS progressively increases over a grafting time of 0 to 4 h, reaching a minimum contact angle of ≈11 degrees (Figure 2C). The spherical organoids formed in the micro‐well chip grafted for 4 h were uniform and regular, indicating that the PEGMA surface could diminish cell attachment and ECM adhesion (Figure 2D,E). The diameters of the spherical organoids were ≈900 µm, accompanied by an aspect ratio of about 0.93 and a Young's modulus of ≈600 Pa (Figure 2F,G). By examining through scanning electron microscopy, it was observed that within liver organoids, hepatocytes and endothelial cells are layered and stacked on top of each other both on the outer and inner layers of the extracellular matrix (Figure 2H). Furthermore, immunofluorescence staining analysis revealed that CD31+ liver sinusoidal endothelial cells are able to connect with each other within the organoid spheres to form a preliminary vascular network, while ALB+ hepatocytes are uniformly distributed within the organoid spheres (Figure 2I). In summary, we contended that PEGMA‐grafted PDMS micro‐well chips exhibit excellent low‐attachment propensity and biocompatibility attributes, rendering them suitable for forming and cultivating 3D liver organoids.

### 2.3 hESCs‐Derived Organoids Achieve Hepatocyte Zonation by WNT Signaling Modulation from Zonated PC and PP LSECs

**Figure 3:**
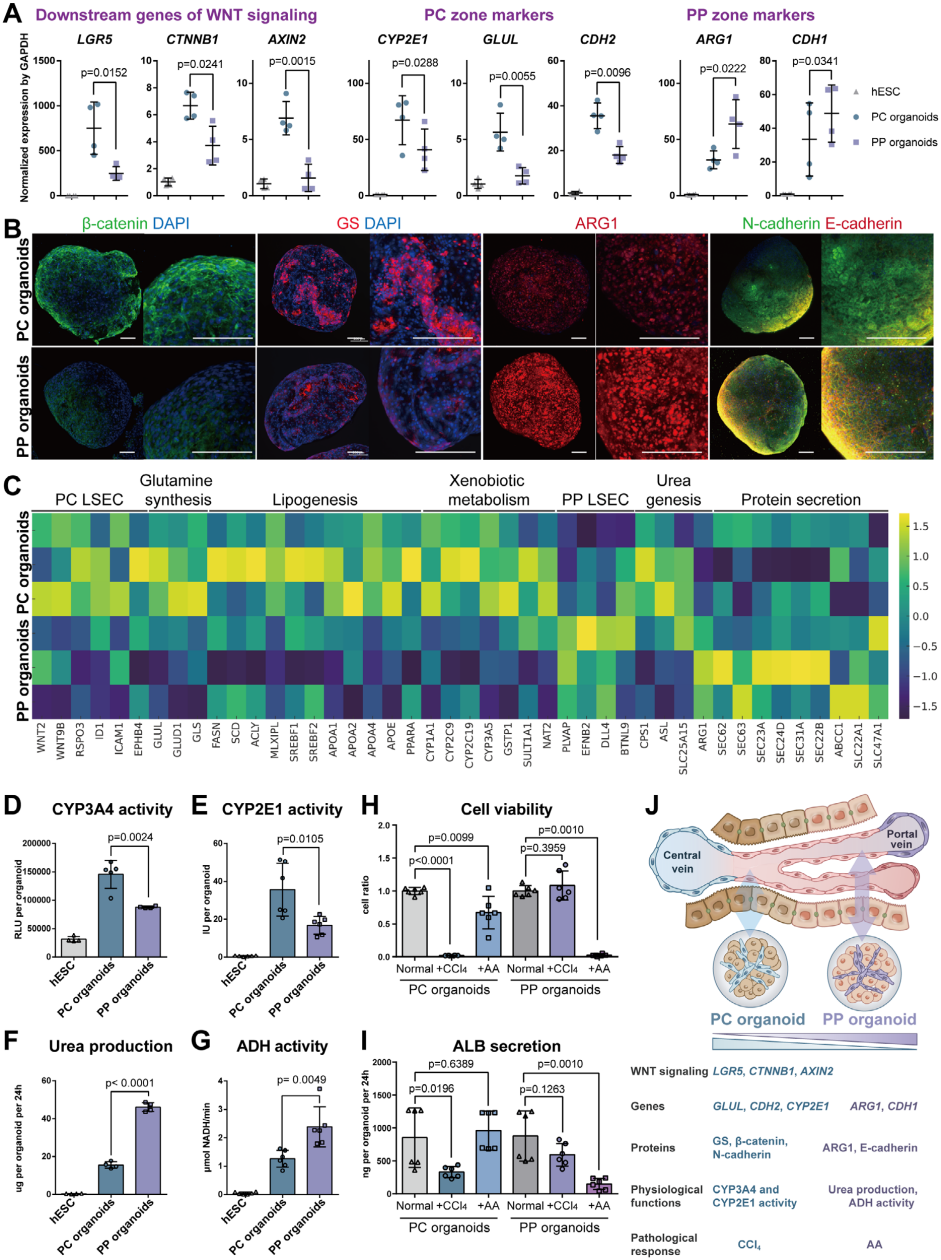
hESCs‐derived organoids achieve hepatocyte zonation. A) qPCR results for the expression of downstream genes of the WNT/β‐catenin signaling pathway (LGR5, CTNNB1, AXIN2), primary liver PC zone markers (GLUL, CYP2E1 and CDH2) and primary liver PP zone markers (ARG1 and CDH1) in PC and PP organoids, with GAPDH as a housekeeping gene. Data analysis was conducted using one‐way ANOVA to compare differences between the PC and PP groups (*n* = 4). B) Immunofluorescence staining of primary PC zone markers (β‐catenin, N‐cadherin) and primary PP zone markers (ARG1, E‐cadherin) for PC and PP organoids, with scale bar of 200 µm. C) RNA sequencing analyzed genes related to in vivo pericentral zone function (glutamine synthesis, lipogenesis, xenobiotic metabolism) and in vivo periportal zone function (urea genesis, protein secretion) of PC and PP organoids. The heatmap displays the results from three replicate experiments. D) Measurement of the CYP3A4 activity for PC and PP organoids. Relative light units (RLU) per organoid is indicated. Significance was determined using one‐way ANOVA, selecting for comparison between PC and PP groups (*n* = 5). E) Measurement of the CYP2E1 activity for PC and PP organoids. The enzyme activity is indicated in International Units (IU) per organoid. Significance was determined using one‐way ANOVA, selecting for comparison between PC and PP groups (*n* = 6). F) Measurement of urea production in PC and PP organoids. Urea amount per organoid is indicated. Significance was determined using one‐way ANOVA, selecting for comparison between PC and PP groups (*n* = 4). G) Measurement of ADH enzyme activity in PC and PP organoids. Enzyme activity levels that expressed as the amount of NADH (in micromoles) produced per min per organoid is indicated. Significance was determined using one‐way ANOVA, selecting for comparison between PC and PP groups (*n* = 6). H) Cell viability assays of PC and PP organoids in medium with 4 mM carbon tetrachloride (CCl_4_) and 100 µM allyl alcohol (AA) for 24h. The absorbance ratios of each group, relative to the control group in normal medium, are indicated. Significance was determined using one‐way ANOVA, selecting for comparison between control and drug treated groups (*n* = 6). I) Albumin secretion level of PC and PP organoids in medium with 4 mM CCl_4_ and 100 µM AA for 24h. Albumin amount per organoid is indicated. Significance was determined using one‐way ANOVA, selecting for comparison between control and drug treated groups (*n* = 6). J) This schematic delineates the differences between PC and PP organoids across five key aspects: WNT signaling, marker gene expression, marker protein expression, physiological function and pathological response.

To investigate whether the phenotype of hepatocytes can be regulated by endothelial phenotype differences through cell‐cell interactions during co‐cultivation, we co‐cultured hESCs‐derived hepatocytes separately with hESCs‐derived PC LSECs and PP LSECs on PDMS microchips to generate PC organoids and PP organoids mimicking in vivo liver PC and PP zone (Figure S5A–C, Supporting Information). Quantitative PCR analysis unveiled differential expression patterns in two types of organoids, showing elevated levels of downstream genes of the WNT2 signaling pathway (*LGR5*, *CTNNB1*, *AXIN2*) and in vivo pericentral markers (*GLUL*, *CYP2E1*, *CDH2*) in PC liver organoids compared to PP liver organoids, while in vivo periportal markers (*ARG1*, *CDH1*) exhibited lower expression in PC liver organoids as opposed to PP liver organoids (**Figure** **3**A). Through immunofluorescence staining analysis of protein expression levels, we found that downstream of the WNT2 signaling pathway (β‐catenin) and in vivo pericentral markers (GS and N‐cadherin) showed higher expression in PC liver organoids compared to PP liver organoids, while in PP liver organoids, in vivo periportal markers (ARG1 and E‐cadherin) exhibited higher expression than in PC liver organoids (Figure 3B). RNA sequencing analysis revealed that PC organoids demonstrated enhanced glutamine synthesis (e.g., *GLUL*, *GLUD1*, *GLS*), lipogenesis (e.g., *PPARA*, *FASN*, *SCD*, *ACLY*, *SREBF*, *APOA*, *APOE*), CYP450 enzyme activity (e.g., *CYP1A1*, *CYP2C9*, *CYP2C19*, *CYP3A5*), and other xenobiotic metabolism (e.g., *GSTP1*, *SULT1A1*, *NAT2*) compared to PP organoids (Figure 3C and Figure S5D, Supporting Information). Conversely, PP organoids showed superior protein secretion (e.g., *SEC23 and 24*, *SEC62 and 63*, *SEC31*, *SEC22*, *ABCC1*, *SLC22*) and ureagenesis, notably in the final step of converting arginine to urea through *ARG1*, in relation to PC liver organoids (Figure 3C and Figure S5D, Supporting Information). In addition, the two types of organoids reveal distinct metabolic differences in hepatocyte function with PC liver organoids exhibiting enhanced enzyme metabolism of CYP3A4 and CYP2E1 compared to PP liver organoids (Figure 3D,E), while PP liver organoids show a superior ability to convert ammonium chloride into urea and alcohol into aldehyde relative to PC liver organoids (Figure 3F,G).

Furthermore, the two types of organoids exhibited variations in damage response under distinct pathological conditions. Since the PC organoid exhibited higher CYP2E1 activity (Figure 3E), it converts carbon tetrachloride (CCl₄) into highly reactive trichloromethyl free radicals (·CCl_3_) and trichloromethyl peroxide (·CCl_3_O_2_), leading to lower cell viability and reduced albumin secretion in PC liver organoids compared to PP liver organoids (Figure 3H,I). While the PP organoid has higher alcohol dehydrogenase (ADH) activity (Figure 3G), converting allyl alcohol (AA) into toxic acrolein, this led to lower cell viability and reduced albumin secretion in PP liver organoids compared to PC liver organoids (Figure 3H,I). In summary, the regulation of liver‐like organogenesis by endothelium exhibited zonal characteristics at the gene/protein expression levels, physiological functions and responses to hepatic toxins that closely resemble those of hepatic cells within the in vivo niche (Figure 3J)

**Figure 4:**
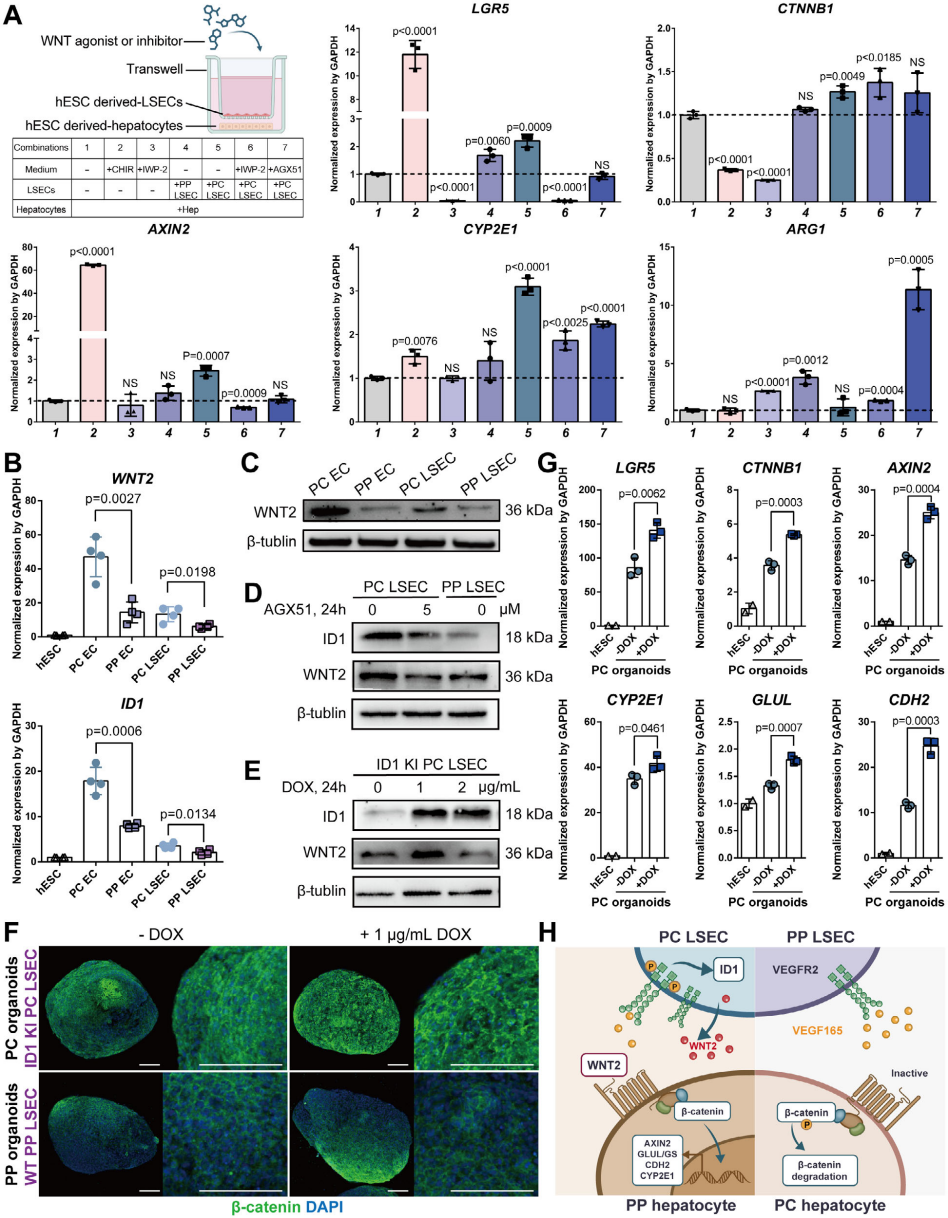
Hepatocyte zonation was modulated by WNT2 signaling from zonated PC and PP LSECs. A) qPCR results for the expression of WNT/β‐catenin signaling pathway (LGR5, CTNNB1, AXIN2), primary liver PC zone marker (CYP2E1) and primary liver PP zone marker (ARG1) in hESCs‐derived hepatocytes that are co‐cultured with hESCs‐derived PC or PP LSECs and small molecular in transwell, with GAPDH as the housekeeping gene. The culture medium was supplemented with three different small molecules: CHIR99021 at 2 µM, IWP‐2 at 5 µM, and AGX51 at 5 µM. Significance was determined using one‐way ANOVA, selecting for comparison between control and the other groups (*n* = 3). B) qPCR results for the expression of WNT2 and ID1 genes in hESCs‐derived PC EC, PP EC, PC LSEC and PP LSEC, with GAPDH as the housekeeping gene. Significance was determined using one‐way ANOVA, selecting for comparison between PC and PP groups (*n* = 4). C) Western blot analysis for WNT2 protein in hESCs‐derived PC and PP LSEC, with β‐tublin as the housekeeping protein. D) Western blot analysis for ID1 and WNT2 protein in hESCs‐derived PC LSEC, PP LSEC and PC LSEC treated with 5 µM ID1 inhibitor AGX51 for 24 h, with β‐tublin as the housekeeping protein. E) Western blot analysis for ID1 and WNT2 protein in ID1 knock in (KI) hESCs‐derived PC LSEC treated with 0, 1, and 2 µg mL^−1^ doxycycline (DOX) for 24 h, with β‐tublin as the housekeeping protein. F) Immunofluorescence staining of β‐catenin for hESCs‐derived PC and PP organoids, with and without treatment with 1 µg mL^−1^ DOX, with scale bar of 200 µm. PC organoids are composed of ID1 KI PC LSECs and WT hepatocytes, whereas PP organoids are composed of WT PP LSEC and WT hepatocytes, with scale bar of 200 µm. G) qPCR results for the expression of downstream genes of the WNT/β‐catenin signaling pathway (LGR5, CTNNB1, AXIN2) and primary liver PC zone markers (GLUL, CYP2E1 and CDH2) in hESCs‐derived PC organoids, with and without treatment with 1 µg mL^−1^ DOX, with GAPDH as the housekeeping gene. PC organoids are composed of ID1 KI PC LSECs and WT hepatocytes. Significance was determined using one‐way ANOVA, selecting for comparison between the group with DOX and the group without DOX (*n* = 3). H) The schematic illustrated the interaction between LSECs and hepatocytes in PC and PP organoids. ID1‐WNT2‐β‐catenin axis may be one of the potential underlying mechanisms by which LSECs regulate hepatocyte zonation in vitro.

To further verify the effect of LSEC secretory signals on hepatocyte zonation, co‐culture experiments of hESCs‐derived LSECs and hepatocytes in a trans‐well system were conducted. It was observed that co‐culture with hESCs‐derived PC LSECs or addition of WNT agonists CHIR99021 led to the upregulation of downstream genes (i.e., *LGR5*, *CTNNB1*, *AXIN2*) of the WNT2 signaling pathway and in vivo pericentral marker genes (*CYP2E1*) in hepatocytes, while co‐culture with hESCs‐derived PP LSECs or addition of WNT inhibitor IWP2 led to the upregulation of in vivo periportal marker genes (*ARG1*) (**Figure** **4**A). Additionally, co‐culturing with hESCs‐derived PC LSECs in the presence of WNT inhibitor IWP2 can also activated the pericentral marker gene *CYP2E1*, suggesting that there exist other factors secreted by LSECs besides WNT signals to regulate the zonation phenotype in hepatocytes (Figure 4A). Regarding the mechanisms underlying the interplay between LSECs and hepatocytes in organoids, since ID1 has been demonstrated to mediate the upregulation of WNT2 signaling in hepatic sinusoidal endothelium during liver regeneration,^[^ ^5^ ^]^ we hypothesize that hESC‐derived LSECs may potentially regulate the zonal phenotype of hepatic cells akin to the in vivo scenario by modulating the secretion of WNT2 signal through the transcription factor ID1. Both gene and protein expression levels of ID1 and WNT2 signaling were upregulated in PC LSECs compared to PP LSECs (Figure 4B,C). To investigate the impact of altered ID1 expression levels on WNT2 expression, we generated conditions of ID1 downregulation using an inhibitor (AGX51) and facilitated ID1 overexpression using a doxycycline‐inducible system in hESCs. Western blot analysis demonstrated that reducing ID1 expression in hESC‐derived endothelial cells by AGX51 resulted in decreased WNT2 expression (Figure 4D and Figure S6A–C, Supporting Information), while inducing ID1 overexpression in hESC‐derived endothelial cells led to heightened WNT2 expression (Figure 4E and Figure S6D,E, Supporting Information). To assess whether low ID1 expression affects the endothelial regulation of hepatocytes, AGX51 was added to the transwell‐based co‐culture of hESC‐derived PC LSECs with hepatocytes. This led to decreased expression of *LGR5* and increased expression of in vivo periportal marker gene *ARG1*, which indicates that lower ID1 expression may cause the ablation of angiocrine WNT signaling, resulting in decreased β‐catenin activation and subsequently perturbed liver metabolic zonation (Figure 4A). To assess whether ID1 overexpression influences endothelial regulation of hepatocyte, PC organoids were generated from ID1 KI hESC‐derived PC LSECs with and WT hESC‐derived hepatocytes. Following 5 days of doxycycline induction, there was a slight increase in the expression of β‐catenin in PC organoids with ID1 KI LSECs, while there was no obvious change in the PP organoids (Figure 4F). Compared to ID1 KI LSECs‐formed PC organoids without DOX, those treated with DOX showed significantly increased expression of *CTNNB1*, *LGR5*, *GLUL*, *AXIN2*, *CDH2*, and slight elevations in *CYP2E1* (Figure 4G). In summary, within our engineered organoid model, hESCs‐differentiated endothelial cells exert regulatory control over the hepatocyte zonation phenotype primarily through the ID1‐WNT2 axis, among other mechanisms (Figure 4H).

### 2.4 Establishment and Application of Zonated Liver MASLD Organoids

**Figure 5:**
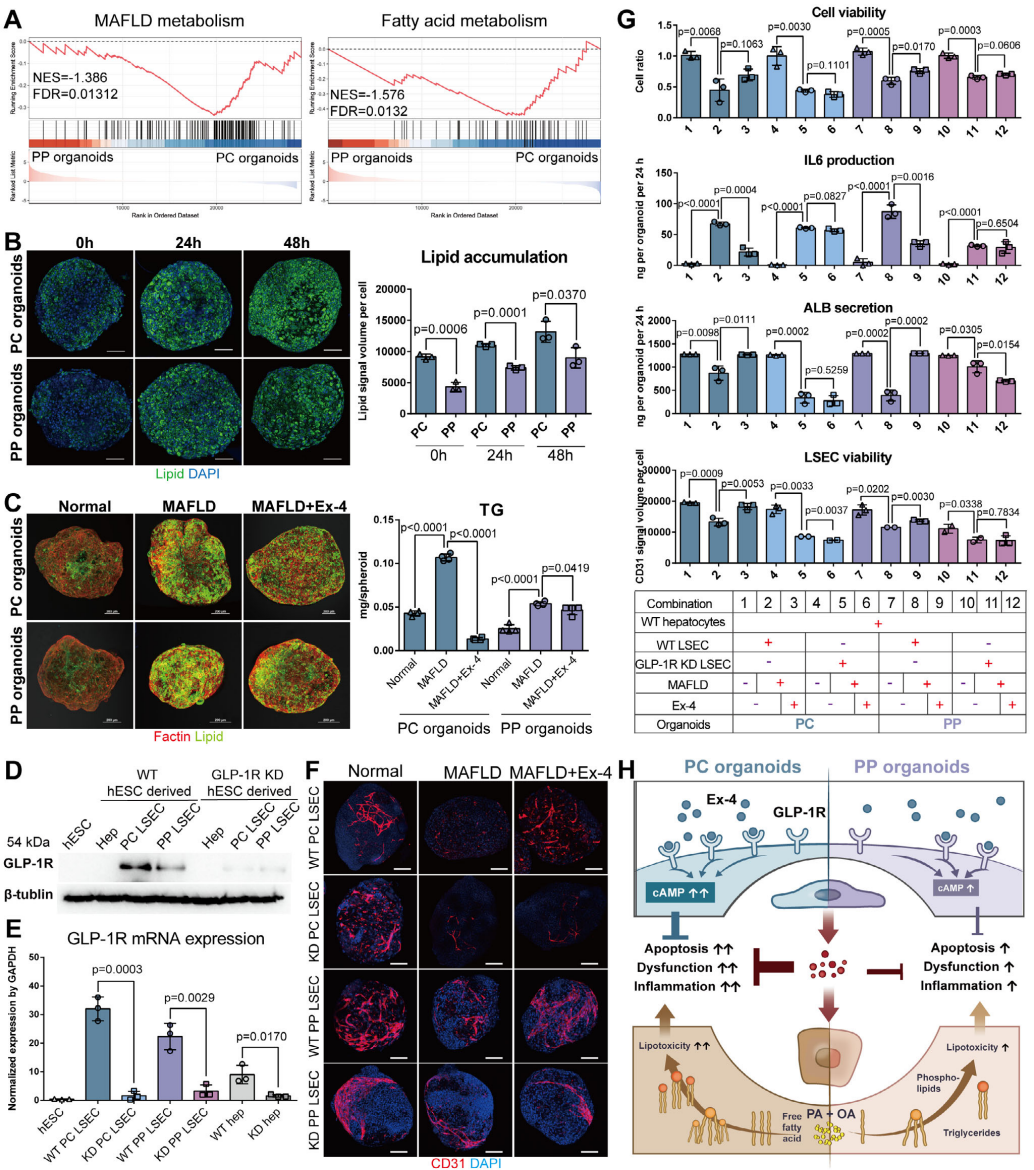
Exendin‐4 alleviates cell apoptosis and inflammation in biomimetic liver zonated PC and PP MASLD organoids through GLP‐1R expressed on zonated PC and PP LSECs. A) Gene set enrichment analysis of MASLD and lipid metabolism pathways in PC and PP Organoids. B) Characterization of lipid accumulation in PC and PP organoids treated with 450 µM OA and 150 µM PA at 0, 24, and 48 h. Lipid accumulation is visualized as green fluorescence signal, quantified by dividing the volume of the green signal by the blue DAPI signal, which stains nuclei. Significance was determined using t‐test on the PC and PP group at each time point (*n* = 3). C) Characterization of lipid accumulation and TG level in PC and PP organoids after treatment with normal culture medium for 5 days, MASLD (450 µM OA and 150 µM PA) culture medium for 5 days, and MASLD culture medium supplemented with 200 nM Exendin‐4 (Ex‐4) for 5 days. Lipid accumulation is visualized in green, while F‐actin, characterizing the cytoskeleton, is depicted in red. In TG level assay, significance was determined using one‐way ANOVA (*n* = 4). D) Western blot analysis for GLP‐1R protein in hESC, WT hESCs‐derived PC LSEC, PP LSEC, hepatocyte (Hep), and GLP‐1R KD hESCs‐derived PC LSEC, PP LSEC, Hep, with β‐tublin as the housekeeping protein. E) qPCR analysis for GLP‐1R protein in hESCs, WT hESCs‐derived PC LSEC, PP LSEC, hepatocyte (Hep), and GLP‐1R KD hESCs‐derived PC LSEC, PP LSEC, Hep, with GAPDH as the housekeeping gene. Significance was determined using one‐way ANOVA, selecting for comparison between the WT group and the KD group for each type of cells(*n* = 3). F) Immunofluorescence staining of CD31 in hESCs‐derived PC and PP organoids treated with Normal, MASLD, MASLD + 200 nM Ex‐4 medium for 5 days. PC organoids are composed of PC LSEC or GLP‐1R KD PC LSECs with WT hepatocytes, whereas PP organoids are composed of PP LSEC or GLP‐1R KD PP LSECs with WT hepatocytes, with scale bar of 200 µm. G) Cell viability assays, IL6 production, albumin secretion and LSEC cell viability of hESCs‐derived PC and PP organoids treated with Normal, MASLD, MASLD + 200 nM Ex‐4 medium for 5 days. PC organoids are composed of PC LSEC or GLP‐1R KD PC LSECs with WT hepatocytes, whereas PP organoids are composed of PP LSEC or GLP‐1R KD PP LSECs with WT hepatocytes. For cell viability assays, the absorbance ratios of each group, relative to the corresponding control group in normal medium, are indicated. Albumin and IL6 amount per organoid are indicated. LSEC cell viability is visualized as a red CD31 fluorescence signal. F), quantified by dividing the volume of the red signal by the blue DAPI signal, which stains nuclei. Significance was determined using one‐way ANOVA, comparing the Normal group to the MASLD group and the MASLD group to the MASLD+Ex‐4 group for each type of organoid sample (*n* = 3). H) The schematic illustrated the potential explanation for the therapeutic mechanism of GLP‐1RA in the treatment of MASLD PC and PP organoids.

Owing to the regulatory influence of WNT on liver lipid metabolism,^[^ ^23^, ^24^ ^]^ notable differences in lipid metabolism were observed between PC organoids and PP organoids. Metabolic pathways, including fatty acid metabolism, biosynthesis of unsaturated fatty acids, PPAR signaling, cholesterol metabolism, CYP450 metabolism, glycerophospholipid metabolism and MASLD metabolism, demonstrated increased activity in PC organoids compared to PP organoids (**Figure** **5**A and Figure S7A, Supporting Information). MASLD is a widespread disease without specific drug treatments. Multiple cell‐based and animal‐based (e.g., mouse model) experiments, as well as clinical trials, have suggested that GLP‐1R could be a potential target for MASLD treatment, although its mechanism of action remains unclear.^[^ ^25^ ^]^ We propose that human zonated MASLD organoids could be utilized to verify the effects of GLP‐1RA drugs and conduct related mechanistic investigation. Initially, MASLD organoids were induced by supplementing the organoid medium with free fatty acids (FFA), consisting of 450 µM oleic acid (OA) and 150 µM palmitic acid (PA) (Figure S7B, Supporting Information). Subsequent observations indicated that PC organoids accumulated lipid droplets at a faster rate compared to PP organoids, and the total amount of lipid droplets accumulated was also greater in PC organoids than in PP (Figure 5B). The addition of 200 µM Exendin‐4 (Ex‐4), a GLP‐1R agonist, into both PC and PP organoids resulted in a modest reduction in the amount of lipid droplets, yet there was a notable decrease in the total amount of triglycerides (TG) contained within them (Figure 5C). It is hypothesized that GLP‐1RA may predominantly enhance the oxidation of fatty acids and may also increase the export of TGs from the liver. Next, we developed a GLP‐1R knockdown (KD) ESC cell line using lentivirus‐mediated shRNA to further investigate the mechanism of GLP‐1RA treatment. Western blot and quantitative PCR results indicated the presence of GLP‐1R in wild‐type (WT) hESCs‐derived PC and PP LSECs, and the absence of GLP‐1R in both WT hESCs‐derived hepatocytes and GLP‐1R KD hESCs‐derived PC and PP LSECs (Figure 5D,E and Figure S7C, Supporting Information). Addition of Ex‐4 to the culture medium activated downstream cAMP signaling.^[^ ^26^ ^]^ Under 2D conditions, WT hESCs‐derived PC LSECs produced more cAMP in response than PP LSECs, while both WT hESCs‐derived hepatocytes and GLP‐1R KD hESCs‐derived PC and PP LSECs showed no response to the agonist (Figure S7C, Supporting Information). Meanwhile, under MASLD conditions induced by FFA, Ex‐4 could reduce apoptosis of WT ESC‐derived PC LSECs and PP LSECs, and had no effect on GLP‐1R KD hESCs‐derived PC and PP LSECs (Figure S7D, Supporting Information).

Furthermore, 3D organoid cultures were utilized using four LSEC subtypes, WT PC, GLP‐1R KD PC, WT PP, and GLP‐1R KD PP, each co‐cultured with WT hepatocytes (Figure 5F). In WT PC and PP LSEC organoids, treatment of Ex‐4 was observed to reduce cellular apoptosis as demonstrated by cell viability assays, improve liver function as evidenced by increased ALB secretion, reduce inflammation through decreased IL‐6 production, and enhance the survival of endothelial cells as revealed by increased CD31 fluorescence signals (Figure 5G). In contrast, in GLP‐1R KD PC and PP LSEC organoids, Ex‐4 did not demonstrate a significant effect (Figure 5G). These comparisons confirmed that GLP‐1RA targets GLP‐1R expressed on LSECs, rather than directly impacting hepatocytes. In conclusion, within the liver, GLP‐1RA is likely to alleviate MASLD indirectly through activation of GLP‐1R on the LSECs, rather than directly affecting hepatocytes (Figure 5H). Zonated organoids thus provide a valuable model for exploring the varied phenotypes of MASLD and assessing the impacts and mechanism of action of various drugs.

## 3 Conclusion

Our work represents the first study to develop hPSC‐derived LSECs with differential WNT2 expression, aligning with zonation‐specific characteristics, and to modulate the emergence of zonal metabolic traits in hepatocytes. This work highlights the crucial role of endothelial cells in regulating liver organoid heterogeneity and mediating the mechanisms of action of GLP‐1RA.

The hESC‐derived LSECs we differentiated, whether directed toward arterial or venous phenotypes, were able to highly express the marker proteins of in vivo human LSECs. This was demonstrated not only through immunofluorescence staining experiments but also using an endogenous LYVE1‐mCherry reporter system. In addition, our protocol, which takes 13 days to yield LSECs, is cost‐effective, with clearly defined components, making it suitable for applications in cell therapy, large‐scale organoid production, and other clinical needs. However, hESC‐derived PP LSECs exhibit notable limitations in replicating in vivo periportal LSEC characteristics, including reduced expression of zonation markers such as EFNB2 and DLL4 (Figure 1E), only modest similarity to their human liver counterparts in Pearson correlation analysis (Figure S1D, Supporting Information), and an inferior cellular state (Figure S5C, Supporting Information). To enhance PP LSEC fidelity, future work must optimize differentiation conditions to better mimic the periportal microenvironment, potentially incorporating extracellular matrix remodeling, shear stress, or hydrostatic pressure alongside VEGF‐independent regulatory factors.

In the cultivation of 3D organoids, commercially available low‐adhesion plates are convenient to use, but they have disadvantages such as being non‐reusable, having unstable low‐adhesion effects, being expensive, and not customizable to laboratory needs. Hinman et al. reported that the PEGMA‐graftment methodology could effectively achieve low adsorption of large molecules such as collagen, proteins, and DNA on PDMS.^[^ ^27^ ^]^ We adopted this method to build a PEGMA‐grafted PDMS microwell chip, which proved more effective for low‐attachment culture of cells and organoids compared to other published methods involving modifications of PDMS with Pluronic F‐127,^[^ ^28^ ^]^ polyvinylpyrrolidone (PVP),^[^ ^29^ ^]^ and bovine serum albumin (BSA)^[^ ^30^ ^]^ (Figure S4, Supporting Information). It should be noted that PEGMA‐graftment might lead to cytotoxicity, thus requiring repeated rinsing of the chip with alcohol and deionized water. This PEGMA‐grafted PDMS microwell chip demonstrates superior capabilities in promoting low cellular adhesion, preserving cell viability, and enabling the formation of organoids. Moreover, its modification allows for repeated usage, withstanding 7–10 cleaning cycles without compromising its functionality (Figure S4, Supporting Information). In terms of plate design, there is a groove that can accommodate 3 µL of liquid, making the loading of cell suspensions into wells more precise and ensuring a more uniform cell distribution. The organoids produced are uniform and controllable, suitable for large‐scale operations. Overall, our design of low‐adhesion plates can provide good reference for laboratories to prepare personalized plates and for the commercial large‐scale production of organoid plates.

In the construction of zonated liver organoids, the basis for achieving zonated liver organoids in vitro depends on the understanding of in vivo liver cell zonation mechanisms. Currently, liver zonation mechanisms mainly include endothelial WNT signaling, the Renin‐Angiotensin System (RAS) signaling ^[^ ^31^ ^]^ and glucagon.^[^ ^32^ ^]^ In our preliminary tests, glucagon could not induce hepatocyte zonation (Figure S8A, Supporting Information), and EGF in the RAS signaling could induce zonation of the *CDH1* gene but not the important *ARG1* gene (Figure S8A, Supporting Information). In our study, endothelial WNT signaling induced hepatocyte zonation more effectively than the two above‐mentioned methods. Moreover, although WNT proteins are secretory, their solubility is poor. Due to the absence of specific small molecule agonists for WNT2, WNT9B and RSPO3, it is currently impossible to directly add small molecules to regulate hepatocyte zonation. Our experiments show that it is possible to induce local zonation characteristics in hepatocytes by adding broad‐spectrum WNT agonists or inhibitors, but the effect is no better than co‐culturing with endothelial cells. For instance, the WNT agonist CHIR99021 increases CYP2E1 gene expression but does not enhance GLUL gene expression (Figure S8A, Supporting Information); the WNT inhibitor IWP2 elevates ARG1 expression but fails to decrease CYP2E1 gene expression (Figure 4A, Supporting Information). Beyond providing signals like WNT2, the significance of endothelial cells in organoids far exceeds that of small molecules, as evidenced by GLP‐1RA studies in MASLD organoids. Notably, GLP‐1R is mainly found on endothelial cells, with minimum expression on hepatocytes, highlighting the critical role of endothelial cells in drug response evaluation. At last, to successfully develop zonated liver organoids in vitro, it is crucial to emphasize that the differentiation state of hepatocytes is fundamental in determining the emergence of zonation characteristics. Immature hepatocytes in the AFP+ALB+CYP3A4‐ALT‐ stage are more easily induced to exhibit zonation characteristics, whereas it is difficult to induce zonation characteristics in mature hepatocytes with different endothelia (Figure 3B,C and Figure S8A, Supporting Information). As a long‐term goal, we aim to construct a liver zonation model with more complex cell types and biomimetic oxygen and nutrient conditions.^[^ ^6^, ^33^ ^]^ For the liver zonation by LSEC modulation, literature reports that endothelium can also regulate the zonation of two additional liver cells (i.e., stellate cells and macrophages).^[^ ^6^ ^]^ Similar to zonation, polarization is a critical attribute of functional hepatocytes, particularly in terms of bile canaliculi formation, essential for proper liver function. Interestingly, the canonical Wnt pathway acts as a key regulator in liver zonation and hepatocyte polarization.^[^ ^34^ ^]^ Endothelial cells can secrete Wnt factors, suggesting that endothelialization may facilitatethe formation of proper hepatocyte polarity. Future studies could provide more systematic evaluation of the functional and the ultrastructural level features of polarized hepatocytes.^[^ ^35^ ^]^ In summary, to construct zonated liver organoids in vitro that mimic in vivo heterogeneity, the role of endothelium cannot be ignored, the differentiation state of various cells is crucial, and more in vivo zonation mechanisms need to be studied.

Based on the MASLD model constructed using zonated liver organoids, it has been proposed that GLP‐1RA drugs may exert their effects in the liver by acting on GLP‐1R located on LSECs. Although the liver is considered to barely express GLP‐1R, Panjwani et al. reported detectable levels of GLP‐1R in the liver, but not in hepatocytes or macrophages, suggesting its presence in blood vessels, bile ducts, neurons, or infiltrating immune cells within the liver.^[^ ^36^ ^]^ Besides, researchers have observed through immunohistochemical staining that GLP‐1R is expressed on liver sinusoids located near the central vein, and its expression increases in MASLD.^[^ ^37^ ^]^ In our investigations, GLP‐1R expression was identified in isolated mouse primary LSECs (Figure S8B, Supporting Information). Our finding underpins the hypothesis that GLP‐1RA could exert a direct effect on the liver,^[^ ^36^ ^]^ thereby suggesting a mechanism for their action beyond the traditional glucoregulatory pathways. This discovery contributes to the evolving understanding of GLP‐1RA's role in hepatic pathology and potentially in the development of GLP‐1R targeted therapy for liver diseases. In addition, experiments also show that addition of Ex‐4 to MASLD liver organoids mainly reduces cell apoptosis, enhances hepatocyte function, and decreases inflammation, but does not significantly clear lipid droplets. It was observed that reducing the concentration of FFA in the culture medium proved more effective than the addition of Ex‐4 in reducing lipid droplet accumulation within hepatocytes following MASLD induction (Figure S8C, Supporting Information). This implies that the treatment of MASLD needs to reduce lipid in the blood, improve the immune environment of the liver, and rescue liver cell functions. For the treatment of many diseases, such as weight rebound, gastrointestinal adverse reactions, thyroid cancer, suicidal depression tendencies etc., GLP‐1R is an excellent drug target, but its side effects may raise concerns. This may be due to the wide variety of cells in the body that express GLP‐1R. We speculate that targeting specific diseases in specific regions for treatment, and precise medication might not only cure the disease but also reduce the occurrence of adverse reactions. In the liver, where the GLP‐1R is mainly expressed in sinusoid, in addition to targeting the islets and other means to reduce blood lipid levels for the treatment of MASLD, GLP‐1RA drugs also need target LSECs to improve the immune environment of the liver and rescue liver cell functions.

## 4 Experimental Section

### Cell Culture and Differentiation

The h1 hESC lines were maintained in mTeSR1 essential medium in a 37 °C incubator at 5% CO2 and 100% humidity and the medium was changed daily. hESC lines and hESC‐derived cells were seeded onto Matrigel‐coated plates. For initiation of cell differentiation, hESCs were seeded at a density of 1.2 × 10ˆ5 cells per well of a 12‐well plate in mTeSR1 essential medium containing 10 µM Y27632.

hESC‐derived endothelial cells induction was optimized based on the method described by Zhang et al.^[^ ^20^ ^]^ employing AATS medium from Professor Na Jie's Laboratory, Tsinghua University. Initially, hESCs were differentiated into mesoderm cells by culturing in AATS medium supplemented with 5 ng mL^−1^ BMP4 for 1 day, followed by an additional 2 days with both 5 ng mL^−1^ BMP4 and 2 µM CHIR99021. For the differentiation into PC EC progenitors, mesoderm cells were dissociated using Accutase, seeded onto Matrigel‐coated plates at a density of 2.3×10^5^ cells per well, and cultured in AATS medium enriched with 10 ng mL^−1^ VEGF, 20 ng mL^−1^ bFGF, and B27 supplement for 3 days. In a parallel process, PP EC progenitors were obtained by seeding similarly treated mesoderm cells at a density of 2.8×10^5^ cells per well and cultivating them in AATS medium with 100 ng mL^−1^ VEGF, 20 ng mL^−1^ bFGF, and B27 minus insulin supplement for 3 days. The maturation of both PC and PP progenitors into LSECs was accomplished by treating the cultures with 5 µM SB431542 for 6 days.

Differentiation of embryonic stem cells into Hepatocytes is divided into four stages. The process began with definitive endoderm differentiation, where hESCs were initially cultured in RPMI1640 medium with 100 ng mL^−1^ Activin A, 3 µM CHIR99021, 1% GlutaMax, and B27 supplement on Day 1. This was followed by a continuation in RPMI1640 containing 100 ng mL^−1^ Activin A, 5 ng mL^−1^ bFGF, and 1% GlutaMax, along with B27 supplement for the next 2 days. From Days 4 to 7, the cells were cultured in SFD medium comprising 75% IMDM, 25% Ham's F12, 0.5% N2 supplement, 1% B27 supplement, 0.05% BSA, and 50 mg mL^−1^ ascorbic acid, supplemented with 100 ng mL^−1^ Activin A and 5 ng mL^−1^ bFGF. The subsequent stage of hepatic endoderm formation, from Days 8 to 13, involved culturing the cells in DMEM with 1 ng mL^−1^ glucose, 1% B27 supplement, 1% BSA, 50 mg mL^−1^ ascorbic acid, 40 ng mL^−1^ bFGF, and 50 ng mL^−1^ BMP4. Differentiation into hepatoblasts occurred over Days 14 to 19 in DMEM with 1 mg mL^−1^ glucose, 1% B27 supplement, 1% BSA, 50 mg mL^−1^ ascorbic acid, 20 ng mL^−1^ OSM, 25 ng mL^−1^ HGF, and 1 µM Dexamethasone. The final maturation into premature hepatocytes was carried out from Days 20 to 31 in DMEM with 5 mg mL^−1^ glucose, supplemented with 1% B27 supplement, 1% BSA, 50 mg mL^−1^ ascorbic acid, 20 ng mL^−1^ OSM, 25 ng mL^−1^ HGF, and 1 µM Dexamethasone.

### Fabrication of PEGMA‐Grafted PDMS Microwell Chip

The mold for the chip was fabricated using PDMS as per the design outlined in Figure S3, Supporting Information. PDMS was prepared by mixing the base and the curing agent in a 10:1 ratio, poured into the mold, and then degassed in a vacuum until all bubbles were eliminated. The mold was then placed in a 65 °C oven for overnight curing. After curing, the PDMS was carefully demolded in 35% alcohol and washed with deionized water overnight. For low adsorption modification of the well plate surface, the method referenced Hinman et al.^[^ ^27^ ^]^ and involved ultraviolet (UV) cross‐linking of PEGMA. This was achieved by adding a PEG crosslinker solution containing 10% weight percentage PEGMA monomer, 0.5% weight percentage benzyl alcohol, and 0.5 mM sodium periodate to PDMS well chips. UV cross‐linking was performed using a UV lamp, pausing every 30 min for degassing and replenishment of the solution. Post‐crosslinking, the mold was thoroughly rinsed with deionized water to remove any unreacted PEGMA, followed by continuous washing on a shaker for 3 days, with daily water changes. Subsequently, the modified gel plates were sterilized in 75% alcohol for 24 h and then exposed to UV light for 3 h to ensure complete evaporation of the alcohol, rendering the plates ready for use.

Before developing the cultivation of organoids on PEGMA‐grafted PDMS microwell chips, this work had explored other published methods to reduce cell attachment on PDMS surface, including treatments with Pluronic F‐127[28], PVP[29], and BSA[30]. The 10% Pluronic F‐127 solution was applied to a UV‐sterilized chip and incubated overnight at room temperature; before use, the solution was aspirated and the chip was allowed to air dry. Similarly, the 2% PVP solution was placed on a UV‐sterilized chip and incubated overnight at room temperature. Prior to use, the chips were washed twice with PBS and once with culture medium, followed by the aspiration of all residual liquids. The 10% BSA solution was also prepared, filtered for sterility, and incubated on a UV‐sterilized chip at 37 °C for 2 h; afterward, the BSA solution was removed. Each chip was subsequently ready for experimental use following the aspiration of the respective solutions after their designated incubation periods.

### 3D Liver Organoids Formation

To construct 3D liver organoids in vitro, day 25 hepatocytes along with PC or PP LSECs derived from hESCs were enzymatically digested into single cells. These cells were subsequently resuspended in an organoid formation matrix at a 1:1 ratio. The matrix was composed of Matrigel (3.35 mg mL^−1^) and type I collagen (1.11 mg mL^−1^), blended in organoid growth medium. This growth medium was a mixture of endothelial cell differentiation medium and hepatocyte medium, containing equal parts AATS medium and DMEM/F12 medium. It was further supplemented with 50 ng mL^−1^ VEGF, 20 ng mL^−1^ bFGF, 40 ng mL^−1^ HGF, 20 ng mL^−1^ EGF, 1% GlutaMax, 1% B27, 1% BSA, and 1% penicillin‐streptomycin (PS). The organoid cell suspension was seeded onto a 48‐well PDMS chip at a density of 30 000 cells per well, with each well receiving 3 µL of the suspension. The chip was incubated at 37 °C for ≈45 min to facilitate matrix gelation. After gelation, organoid growth medium supplemented with 10 µM Y27632 was added to each well, and the medium was refreshed daily. The cultured organoids were maintained at 37 °C in a controlled environment of 5% CO2 and 95% air. Organoids, after 5 days of formation, are suitable for a diverse range of analyses, including immunofluorescence, quantitative PCR, atomic force microscopy, scanning electron microscopy, RNA sequencing, and other techniques.

### Construction of LYVE1‐mCherry Reporter hESC Cell Line

The Cas9(sgRNA) plasmid used was pSpCas9(BB)‐2A‐GFP (Addgene plasmid ID: 48 138), and the repair template plasmid was pL552 (Addgene plasmid ID: 68 407), following the methodology outlined by Ran et al.^[^ ^38^ ^]^ The CRISPR Design Tool (http://tools.genome‐engineering.org) was employed to design sgRNAs targeting the C‐terminus of the LYVE1 gene, which were inserted into the PX458 plasmid. The target sequences for designed sgRNAs were 5′‐GAGGCTGGTTTCTTTCATGC‐3′ and 5′‐GAAATGAGGAGACACACCTG‐3′. The homology‐directed repair (HDR) template was constructed by amplifying homologous arm fragments from h1ESC's genomic DNA, extending 1 kb from each side of the stop codon. These two homologous arm fragments were fused with mCherry, LoxP‐PGK‐Hyg‐LoxP through high‐fidelity enzyme‐mediated overlap extension PCR and inserted into the pL552 plasmid. H1 ESCs were transfected with these two plasmids using Lipofectamine 3000. GFP‐positive cells, indicative of successful transfection, were isolated via flow cytometry 48 h post‐transfection. These cells were then cloned and expanded in a culture medium containing 50 µg mL^−1^ hygromycin (Hyg) for 2 weeks. PCR and flow cytometry assessments were conducted to confirm successful gene editing. PCR primers are 5‘‐ AGAGTAGGTCTCAAGGCCAT‐3′ (upstream) and 5‘‐ CTCTCAGTCCCTTCTTGTTGG‐3′ (downstream).

Following confirmation, the cells were further transfected with pCAG‐Cre: GFP (Addgene plasmid ID:13 776) to facilitate recombination and removal of the Hyg segment. The final LYVE1‐mCherry reporter hESC cell line was cultured in mTeSR medium.

### Construction of ID1 Overexpression hESC Cell Line

To establish a Dox‐controlled ID1 overexpression system in hESCs at the AAVS1 (PPP1R12C) site, the first step involved extracting total RNA from endothelial cells differentiated from hESCs. This RNA was reverse transcribed to obtain total cDNA, which served as a template. Using primers 5‘‐TCGGTACCGCCACCATGAAAGTCGCCAGTGGCAG‐3′ (forward) and 5‘‐TCGTCGACAAGCTTATCAGCGACACAAGATGCGAT‐3′ (reverse), the DNA encoding fragment corresponding to ID1 transcript variant 1 was amplified. This fragment was integrated into the pAAVS1‐Neo‐CAG‐M2erTA backbone (Addgene plasmid ID:85 798), which contains a G418 resistance gene and Tet‐on system, through a double enzyme digestion and ligation process (KpnI and SalI), forming the donor plasmid targeted to the AAVS1 site. The CRISPR/Cas9 plasmid used in the experiment was a commercial plasmid (Addgene plasmid ID:118 414) obtained by inserting the sgRNA (5‘‐gTGGGGGTTAGACCCAATATC‐3′) into the pX458 backbone. H1 ESCs were transfected with these two plasmids using Lipofectamine 3000 and selected with 50 µg µL^−1^ G418 commenced 72 h post‐transfection and continued for 2 weeks. Single clones were picked from the wells, expanded in 48‐well plates, and subjected to genomic PCR and sequencing for identification of correctly inserted positive clones, using primers 5‘‐TCGTCGACTCAGCGACACAAGATGCGAT‐3′ (forward) and 5‘‐CTCTGGCTCCATCGTAAGCA‐3′ (reverse).

### Construction of GLP‐1R Knockdown Expression hESC Cell Line

The specific shRNA targeting GLP‐1R was sourced from the MISSION shRNA Library. This shRNA was then cloned into the pLV‐CMV‐puro lentivirus mammalian expression vector, preparing it for effective delivery into hESCs. Lentivirus production was initiated by cotransfecting 293FT cells with this shRNA‐laden expression vector and the second‐generation lentivirus packaging vectors, pVSVG and pΔ8.9. Upon harvesting the lentiviral suspension, it was introduced into h1ESCs seeded in a six‐well plate, utilizing polybrene (8 µg mL^−1^) to enhance viral infection efficiency. To isolate and enrich for cells that successfully integrated the shRNA construct, we added puromycin at a concentration of 2.5 µg mL^−1^ to the culture medium, 24 h post‐infection.

### Induction of MASLD Organoids

After 5 days of formation, organoids can also be utilized to induce MASLD organoids, which were induced by supplementing the organoid medium with FFA. For the preparation of FFA solutions, palmitic acid (PA) and oleic acid (OA) were processed using immediate‐use protocols.

PA solution was prepared by dissolving 0.4 g of NaOH in 100 mL of deionized water to create a 0.1 M NaOH solution, into which 0.0256 g of PA was added. This mixture was then heated in a 75 °C water bath, yielding a 40 mM PA solution. Concurrently, 1.2 g of BSA was dissolved in 2.5 mL of water and centrifuged at 8000 rpm for 5 min without shaking to form a 40% BSA solution, which was preheated to 55 °C. The PA and BSA solutions were mixed in a 1:3 ratio, resulting in a 10 mM PA stock solution, which was then filtered through a 0.22 µm filter. OA solution was similarly prepared by adding 31.7 µL of OA to 2 mL of 0.1 M NaOH, obtaining a 50 mM OA solution. This was followed by the dissolution of 1.8 g of BSA in 5 mL of water and centrifugation at 8000 rpm for 5 min to achieve a 30% BSA solution. A 1:1 mixture of the OA and BSA solutions produced a 25 mM OA stock solution, which was also subjected to 0.22 µm filtration. Both lipid stock solutions were stored at 4 °C.

### RNA Isolation and Reverse Transcription‐PCR

RNA was extracted from both 2D cells and 3D organoids using TRIzol Reagent (Invitrogen). For reverse transcription, 500 ng of the extracted RNA was processed using the Hiscript II qRT SuperMix Kit (V) (Vazyme), converting RNA into complementary DNA (cDNA). Quantitative analysis of gene expression was performed via real‐time PCR, utilizing AceQ qPCR SYBR Green Master Mix (Vazyme) on a CFX96 Real‐Time System (Bio‐Rad). The relative expression levels of target genes were calculated employing the 2^ΔΔCt method, with normalization against the housekeeping gene GAPDH to account for variations in cDNA input across samples. Detailed sequences of the primers used for amplification are provided in Table S1, Supporting Information.

### Immunofluorescence Staining

Cells and organoids were fixed with 4% paraformaldehyde (PFA) for 15 min, ensuring structural preservation, and then washed three times with phosphate‐buffered saline (PBS) to remove any residual fixative. For permeabilization, a 0.5% Triton X‐100 solution in PBS was applied for 20 min, facilitating antibody access to intracellular targets. Subsequently, cells were blocked with 5% bovine serum albumin (BSA) in PBS for 1 h to minimize nonspecific antibody binding. Overnight incubation at 4 °C with primary antibodies, previously diluted in blocking solution, was followed by several washes to remove unbound antibodies. Cells were then incubated with suitable secondary antibodies, prepared as per the primary antibodies' specifications. Cell nuclei were stained with Hoechst 33 342 for 20 min. Images were captured with a Nikon A1 HD25 confocal microscope. The fluorescence intensity of organoids and 2D cells was analyzed using NIS‐Elements software version 5.2.

### Flow Cytometry

To obtain suspended cells, hESC‐derived PC or PP EC and LSECs were digested with accutase at 37 °C for 5 min. Following digestion, the cells were suspended and incubated with primary antibodies at 37 °C for 30 min. The cells were then centrifuged, the supernatant discarded, and the pellet resuspended in fresh culture medium. Subsequently, cell sorting was performed using a BD Influx sorter, and analysis was carried out with a BD Biosciences LSR Fortessa Cell Analyzer. Compensation was adjusted using single‐color controls. Data analysis and result plotting were conducted using FlowJo software.

### mRNA Sequencing and Analysis

Total RNA from 2D cells and liver organoids was extracted using TRIzol and sent to Anoroad Gene Technology Corporation for sequencing. Quality was checked with a KaiaoK5500 Spectrophotometer and RNA Nano 6000 Assay Kit on a Bioanalyzer 2100. Sequencing libraries were made using the NEBNext Ultra RNA Library Prep Kit for Illumina, following the provided instructions. Data cleaning was performed with Trimmomatic to remove low‐quality reads and adaptors. HISAT2 was used for aligning clean reads to the human genome. Gene counts were done with HT‐seq and normalized as RPKM using Ensembl annotations and the edgeR package. DESeq2 identified differentially expressed genes. Functional enrichment analyses for GO and KEGG were conducted with the GOstats package. GSEA used the pre‐ranked module in GSEA 4.03 software. mRNA sequencing for PC EC, PP EC, PC LSEC, PP LSEC and PC and PP organoids was conducted in three independent replicates. Following sequencing, comprehensive gene clustering and Gene Set Enrichment Analysis (GSEA) were performed to identify distinct gene expression pattern.

### AcLDL Uptake

hESCs‐derived PC or PP EC and LSECs were seeded at a density of 2.5 × 10^5^ cells per well in 12‐well plates. Cells were incubated with 10 µg mL^−1^ of Dil‐labeled acLDL (1,1′‐dioctadecyl‐3,3,3′,3′‐tetramethylindocarbocyanine perchlorate‐labeled acetylated LDL) for 4 h at 37 °C in a 5% CO_2_ atmosphere. Post‐incubation, cells were washed thrice with PBS to remove unbound acLDL. Cellular uptake of Dil‐acLDL was quantified by measuring fluorescence intensity using a BD Biosciences LSR Fortessa Cell Analyzer set at an excitation wavelength of 514 nm and an emission wavelength of 565 nm.

### Glucose Metabolism Analysis

To analyze glucose metabolism, this work employed a non‐targeted LC/MS approach following metabolite extraction from cultured cells. Initially, cells were washed with PBS and lysed in cold 80% methanol at −80 °C for an hour, ensuring thorough metabolite recovery. After scraping and collecting the cell lysate, it was centrifuged to separate the supernatant, which was then use N2 to dry at room temperature and stored at −80 °C until analysis.

### Contact Angle Measurement

Contact angle measurement was performed according to ASTM D5946‐17 despite that a droplet of 2 µL was suspended. During measurement, a humidifier was used to stabilize relative humidity at 40% and the ambient temperature was 21 °C. Flat PDMS samples with different grafting time were tested using the sessile drop method on OCA15Pro (Dataphysics, Germany). Deionized water was suspended on the sample and images of the water droplet was captured after the droplet was stable. Images were processed by SCA20 (Dataphysics, Germany) and Yong‐Laplace fitting was used in all images to measure the contact angle. Each sample was tested by at least 4 droplets.

### Organoid Characterization

For scanning electron microscopy (SEM) analysis, liver spheroids were fixed with 2.5% glutaraldehyde in PBS at room temperature for 2 h and subsequently washed three times with PBS. The samples underwent dehydration through treatment with ethanol solutions at increasing concentrations (70%, 80%, 90%, and then 100% twice), each stage lasting for 10 min. After the removal of excess ethanol, the samples were covered with tert‐butanol and stored at 4 °C. They were then freeze‐dried using the Tousimis Samdri‐795 and coated with a 30 nm gold layer using a Quorum Q150T ES sputter coater. Electron microscopy was conducted using a Quanta 200 at an acceleration voltage of 5 keV and a working distance of 5 mm.

Atomic force microscopy (AFM) was utilized to measure the Young's modulus, adhering to the same protocol detailed in our previously published work.^[^ ^20^ ^]^

### Liver‐Specific Function Assay

Following exposure to 2 mM ammonium chloride for 24 h, urea levels in organoid culture media were quantified by employing the Urea Assay Kit. 24 h after replacing the culture medium with fresh medium, collect the supernatant to measure albumin using an Albumin Detection Kit (enzyme‐linked immunosorbent assay) and to measure IL‐6 using an IL‐6 ELISA Kit. After replacing with fresh culture medium, CYP3A4 activity was tested by P450‐Glo CYP3A4 Assay Kit. After losing the organoids by sonication in the culture medium, the supernatant was collected for CYP2E1 detection using a CYP2E1 ELISA Kit. After the organoids are lysed by sonication with the reagents provided by the kit, the supernatant is collected to indirectly quantify ADH activity by monitoring the production of NADH using the ADH detection Kit. After the organoids are lysed by sonication with Reagent I, provided by the kit, triglycerides in the sample undergo hydrolysis. The resultant dye reagents are then oxidized, yielding colored compounds. This process employs the Triglyceride Assay Kit. All of procedures were executed in strict adherence to the manufacturer's instructions.

### Cell Viability Evaluations

Cell viability was assessed by ATP quantifications using the CellTiter‐Glo Luminescent Cell Viability Assay. Luminescence signals were measured using PerkinElmer EnSight Multimode Plate Reader.

### Analysis of Publicly Available Single‐Cell Sequencing Datasets

The single‐cell sequencing dataset used was sourced from Aizarani et al.^[^ ^19^ ^]^. Diffusion pseudo‐time (dpt) analysis.^[^ ^39^ ^]^ was performed, and diffusion maps were generated utilizing the destiny R package. The parameter for the number of nearest neighbors, denoted as k, was configured to 100. Subsequently, Self‐Organizing Maps (SOM) were created using the FateID package, relying on the computed ordering by dpt as the input. The SOM analysis exclusively considered genes with counts exceeding 2 after size normalization in at least one cell. To outline smooth zonation profiles, a local regression was applied to normalized transcript counts following the ordering of cells by dpt. Following this, a one‐dimensional SOM comprising 200 nodes was computed based on these profiles after z‐transformation. In cases where the Pearson's correlation coefficient of the average profiles for neighboring nodes surpassed 0.85, these nodes were merged. The resultant aggregated nodes constitute the gene modules illustrated in the SOM Figures. We employed the plot‐expression function in the FateID package to plot pseudo‐temporal expression profiles for genes that are representative in different regions. The normalized expression data was filtered to only include the genes with the minimum expression of 2 in at least one cell. The pseudo‐time ordering for endothelial cells was obtained using the same approach depicted above by retrieving the eigenvectors of the diffusion map. Outliers were removed to show the trend of gene expressions.

### Pearson Correlation Analysis

Bulk RNA‐seq datasets (anno_1.xlsx, anno_2.xlsx, and anno_3.xlsx) containing gene‐level read counts for four groups (PC EC, PP EC, PC LSEC, and PP LSEC) were processed by removing duplicate gene entries, filtering out genes lacking valid identifiers, and excluding genes with missing values across all groups. For single‐cell RNA‐seq (scRNA‐seq) analysis, we utilized an integrated Seurat object (Seurat v5.2) from Andrews et al.^[^ ^40^ ^]^ selecting major liver cell populations based on UMAP‐annotated clusters, including cholangiocytes, central vein LSECs, portal endothelial cells, hepatocyte subpopulations, and mesenchymal cells. To facilitate direct comparison, we identified a common gene set shared by both bulk and scRNA‐seq datasets. Subsequently, we calculated Pearson correlation coefficients between bulk RNA‐seq (PC EC, PP EC, PC LSEC, PP LSEC) and the average gene expression profiles of each scRNA‐seq subset, quantifying similarity using the squared correlation coefficient (R^2^), averaged across datasets. Analyses were conducted using R (v4.3.1), leveraging packages such as tidyverse, readxl, dplyr, and Seurat for data preprocessing, with heatmaps generated via base R functions.

### Quantification and Statistical Analysis

Statistical analysis was performed using GraphPad Prism 9.0 software. For all experiments in this paper, each dataset was first subjected to tests for normal and lognormal distributions. For univariate data involving two groups, a t‐test was conducted. Data that passed the normality test assumed a Gaussian distribution of residuals and used a parametric test, whereas data that did not pass the normality test did not assume a Gaussian distribution and used a nonparametric test. For univariate samples involving three or more groups, a one‐way ANOVA was performed; for samples with two variables involving three or more groups, a two‐way ANOVA was conducted. Data that passed the normality test assumed a Gaussian distribution of residuals for ANOVA, while data that did not pass used a nonparametric test. Multiple comparisons involved comparing the mean of each group with the means of every other group or with the mean of the control group. All ANOVA and t‐test analyses were unpaired, with statistical differences denoted by *p*‐values marked on each dataset and data expressed as mean ± SEM. Specifics regarding sample sizes and statistical tests are provided in the figure legends.

## Conflict of Interest

The authors declare no conflict of interest.

## Author Contributions

Y.D. and Y.Z. conceived and designed the experiments; Y.Z. performed the experiments and data collection; C.H. assisted with exploring the ID1/WNT2 mechanism; L.S. assisted with WB experiments; L.Z. assisted with designing the PDMS micro‐well chip; Y.N. and X.Z. assisted with bioinformatics data analysis; K.L. assisted with schematics design and production; P.Z. assisted with the liver organoids AFM test; B.W. assisted with the induction of MAFLD organoids; J.W. assisted with gene editing; Z.L., P.Z., and J.N. contributed to project discussions; Y.Z. and Y.D. wrote the manuscript; Y.D. is the principal investigator of the supporting grants.

## Supporting information



Supporting Information

## Data Availability

The data that support the findings of this study are available from the corresponding author upon reasonable request.
