# Correction to “hESCs‐derived Organoids Achieve Liver Zonation Features through LSEC Modulation”

**DOI:** 10.1002/advs.202511802

**Published:** 2025-07-22

Y. Zhang, C. Huang, L. Sun, L. Zhou, Y. Niu, K. Liang, B. Wu, P. Zhao, Z. Liu, X. Zhou, P. Zhang, J. Wu, J. Na, Y. Du, hESCs‐derived Organoids Achieve Liver Zonation Features through LSEC Modulation. Adv. Sci. 2025, 12, e2411667. https://doi.org/10.1002/advs.202411667

In the original version of the article, two issues were identified in Figures S4B and S8C of the Supporting Information.

In Figure S4B (Supporting Information), The image for the “2% PVP” condition was inadvertently duplicated from the “10% BSA” condition during figure assembly. This error has now been corrected, and the revised figure is shown below.


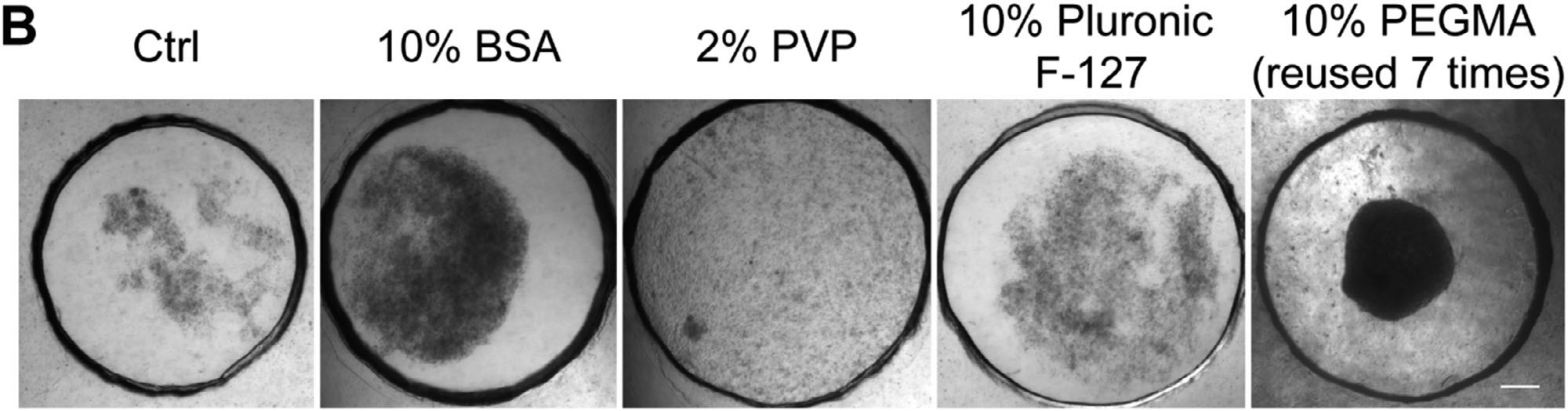


In Figure S8C (Supporting Information), where the three repeated images in the far‐right lane actually represent the same image under identical conditions (“0 µM OA + 0 µM PA”), denoting the normal condition. It serves as the shared control for all MAFLD groups on the left. To avoid potential misunderstanding, we have added an explanatory note to the figure legend. Additionally, the label “NAFLD” in Figure S8C has been corrected to “MAFLD” to reflect the appropriate terminology.

Revised figure legend for Figure S8C: C, D) Characterization (C) and quantification (D) of lipid accumulation in 2D hepatocyte co‐culture with LSEC treated with various concentrations of FFAs and FFAs plus 200 nM Ex‐4. Lipid accumulation is visualized in green, while F‐actin, characterizing the cytoskeleton, is depicted in red. “Minus MAFLD” refers to the use of lower concentrations of FFAs in adjacent conditions. The far‐right column “0 µM OA + 0 µM PA” represents the normal condition, serving as the shared control for all MAFLD groups on the left.


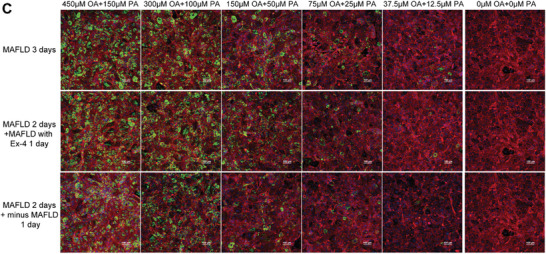


We sincerely apologize for the oversight in Figure S4B and any potential confusion caused by Figure S8C. These errors do not affect the validity of our experimental findings and the conclusions of the study.

