# Supplementary material for: hESCs‐derived Organoids Achieve Liver Zonation Features through LSEC Modulation
Source: Adv Sci (Weinh). 2025 Apr 25;12(20):2411667. doi: 10.1002/advs.202411667 (PMC12120767; doi:10.1002/advs.202411667)
Supplement: Supplementary file 1 — Supporting Information [file ADVS-12-2411667-s001.pdf]

ADVANCED SCIENCE
Open Access

Supporting Information

for *Adv. Sci.*, DOI 10.1002/advs.202411667

hESCs-derived Organoids Achieve Liver Zonation Features through LSEC Modulation

*Yuying Zhang, Chenyan Huang, Lei Sun, Lyu Zhou, Yudi Niu, Kaini Liang, Bingjie Wu, Peng Zhao, Zhiqiang Liu, Xiaolin Zhou, Peng Zhang, Jianchen Wu, Jie Na and Yanan Du**

Supporting Information

hESCs-derived Organoids Achieve Liver Zonation Features Through LSEC Modulation

Yuying Zhang[1,3], Chenyan Huang[4], Lei Sun[1], Lyu Zhou[1], Yudi Niu[1], Kaini Liang[1], Bingjie Wu[1], Peng Zhao[1], Zhiqiang Liu[1], Xiaolin Zhou[5], Peng Zhang[6], Jianchen Wu[3], Jie Na[3], Yanan Du[1, 2, 7]

1 School of Biomedical Engineering, Tsinghua-Peking Center for Life Sciences, Tsinghua University, Beijing, 100084, China.
2 National Key Laboratory of Kidney Diseases
3 School of Basic Medical Science, Tsinghua Medicine, Tsinghua University, Beijing, 100084, China.
4 Department of Molecular Biology, Princeton University, Princeton, NJ, 08544, USA.
5 Institution of Medical Science, University of Toronto, Toronto, Ontario, M5S1A8, Canada.
6 Beijing Children's Hospital, Capital Medical University, Beijing, 100045, China.
7 Lead contact

*Correspondence: Y.D. (email: duyanan@tsinghua.edu.cn)

Table of contents

Figure S1. Comparative gene expression profiling of primary human PC LSECs and PP LSECs reveals differential endothelial identity, related to Figure 1.

Figure S2. Characterization of hESC-derived PC/PP ECs and PC/PP LSECs, related to Figure 1.

Figure S3. Microwell chip and cellular materials for 3D culture, related to Figure 2.

Figure S4. Comparison of methods to promote organoid formation on PDMS micro-well chips, related to Figure 2.

Figure S5. Timeline of PDMS microwell chip fabrication, zonated LSEC and hepatocyte differentiation, and 3D organoid formation, related to Figure 3.

Figure S6. The correlation between ID1 and WNT2, related to Figure 4.

Figure S7. Results related to Figure 5.

Figure S8. Results related to conclusion.

Table S1. Primers for qPCR.

Table S2. Key resources.

Figure S1 Comparative gene expression profiling of primary human PC LSECs and PP LSECs reveals differential endothelial identity, related to Figure 1.

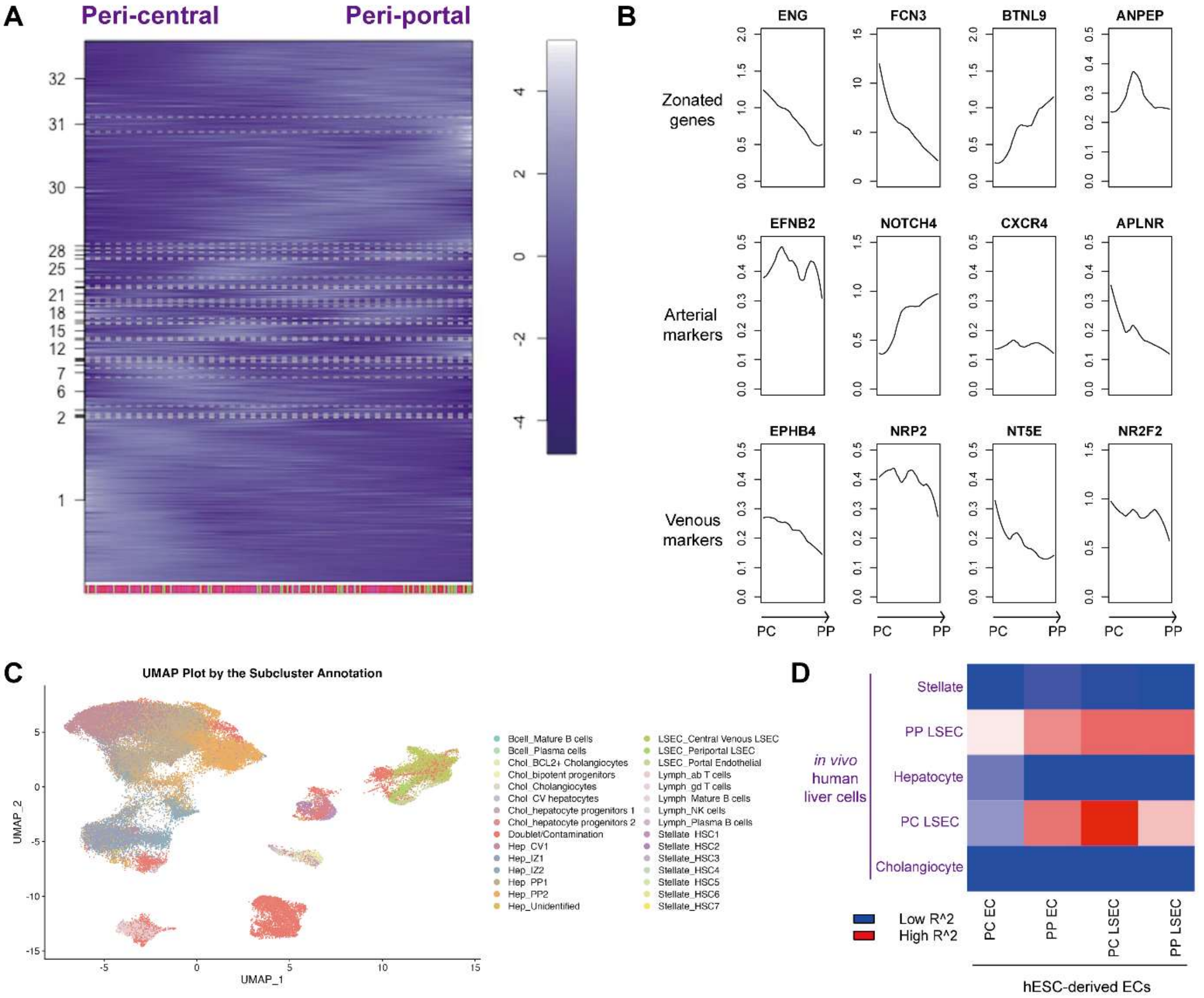

A) Self-Organizing Maps of single-cell transcriptome-derived zonation profiles for endothelial cells (n = 1,361 cells). Colour bars at the right of SOMs show RaceID3 cluster. The gene module pattern is similar to reference paper [1]. **B)** Zonation profiles of LSEC zonation related genes (ENG, FCN3, BTNL9, ANPEP), arterial markers (EFNB2, NOTCH4, CXCR4, APLNR), and venous markers (EPHB4, NRP2, NT5E, NR2F2). The y axis of the zonation profiles indicates normalized expression. **C)** UMAP plot of single-cell transcriptome profiles displaying the assigned identity for each subcluster. scRNA-seq data from published paper [2]. *In vivo* human PC LSEC (n = 1,552 cells), *in vivo* human PP LSEC (n = 340 cells). **D)** Heatmap of Pearson correlation between bulk RNA-seq from hESCs derived ECs and scRNA-seq from human liver samples. The heatmap shows R^2 values for different liver cell types across endothelial compartments. Rows represent *in vivo* cell types, and columns represent hESC-derived endothelial subsets. Colors indicate R^2 values, with blue for low and red for high.

Figure S2 Characterization of hESC-derived PC/PP ECs and PC/PP LSECs, related to Figure 1.

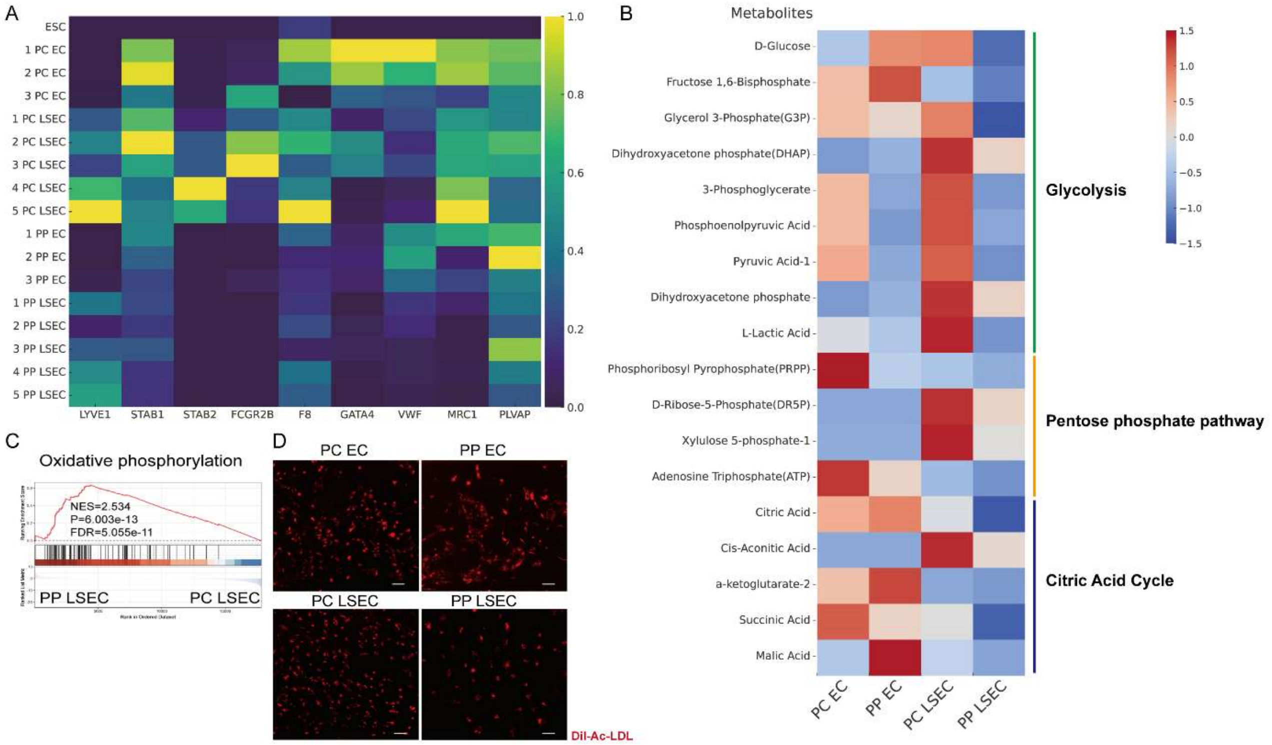

A), RNA sequencing analyzed the expression of human LSEC marker genes for hESC-derived PC/PP ECs and PC/PP LSECs, and the heatmap displays the average results from three replicate experiments. **B)**, Metabolic profiling reveals differential glycolysis, pentose phosphate and tricarboxylic acid cycle metabolite activity in Characterization of hESC-derived PC/PP ECs and PC/PP LSECs. Using mass spectrometry-based metabolomics, we analyzed the concentration of key glucose metabolites in PC ECs and PP LSECs compared to PP ECs and PP LSECs. PC EC and PC LSEC displayed significantly elevated levels of glycolytic and PPP intermediates, including glycerol-3-phosphate(G3P), fructose-6-phosphate (F6P), lactate, glucose-6-phosphate (G6P), phosphoribosyl pyrophosphate (PRPP), deoxyribose-5-phosphate (DR5P) and others, indicative of heightened glycolytic and PPP flux compared to PP EC and PP LSEC. **C)**, Gene set enrichment analysis of Oxidative phosphorylation pathways in hESC-derived PC LSEC and PP LSEC. **D)**, Immunofluorescence image of cell phagocytosing DiI-Ac-LDL in hESC-derived PC/PP ECs and PC/PP LSECs, with scale bar of 50 μm.

Figure S3 Microwell chip and cellular materials for 3D culture, related to Figure 2.

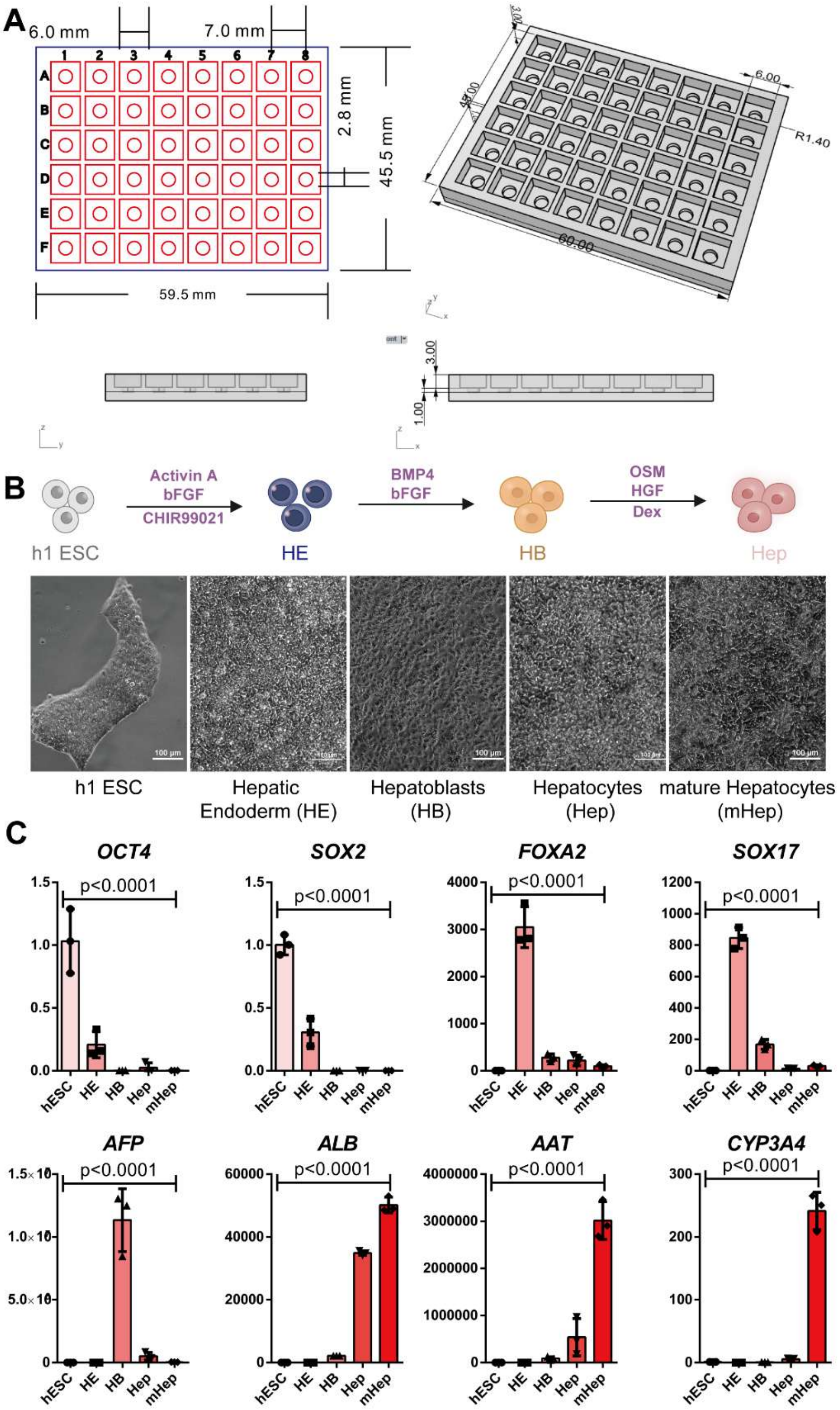

A), Schematic and three-dimensional diagrams of the cell culture plate, with specific dimensional parameters labeled on the illustrations. **B),** The differentiation process of hepatocytes and the morphological changes at each stage. **C),** qPCR Analysis of hepatocyte differentiation markers. OCT4 and SOX2 for hESC, FOXA2 and SOX17 for hepatic endoderm (HE), AFP for hepatoblasts (HB), ALB for hepatocytes (Hep), and AAT and CYP3A4 for more mature hepatocytes (mHep). Data analysis was conducted using one-way ANOVA to compare overall differences (n=3).

Figure S4 Comparison of methods to promote organoid formation on PDMS micro-well chips, related to Figure 2.

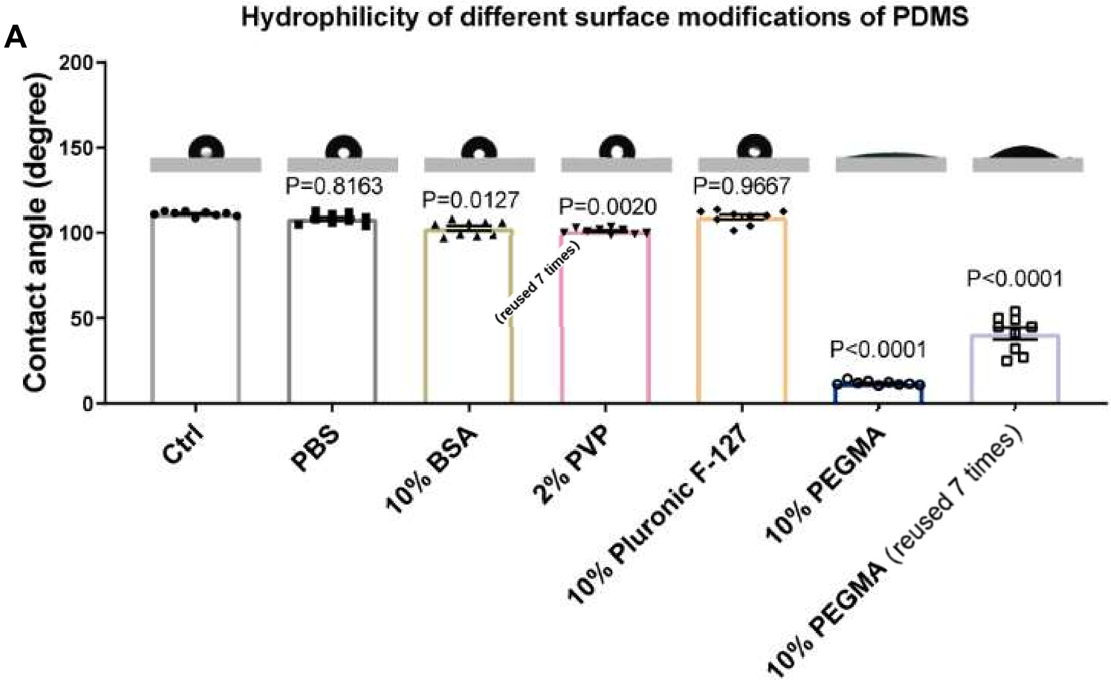

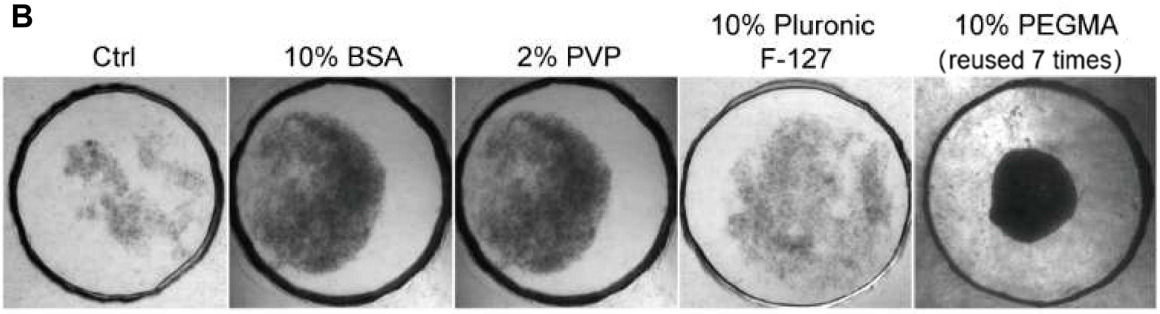

A), Contact angle measurement of PDMS surfaces treated with various modification methods, such as 10% BSA, 2% PVP, 10% Pluronic F-127 and 10% PEGMA. Measurements were conducted in accordance with ASTM D5946-17, using a 2 µL water droplet. Significance was determined using one-way ANOVA, selecting for comparison between control and each modification group (n=6). **B),** Enlarged bright-field images showing organoid formation 48 h after cell seeding on various modified PDMS surfaces, with a scale bar of 500 µm.

Figure S5 Timeline of PDMS microwell chip fabrication, zonated LSEC and hepatocyte differentiation, and 3D organoid formation and characterization, related to Figure 3.

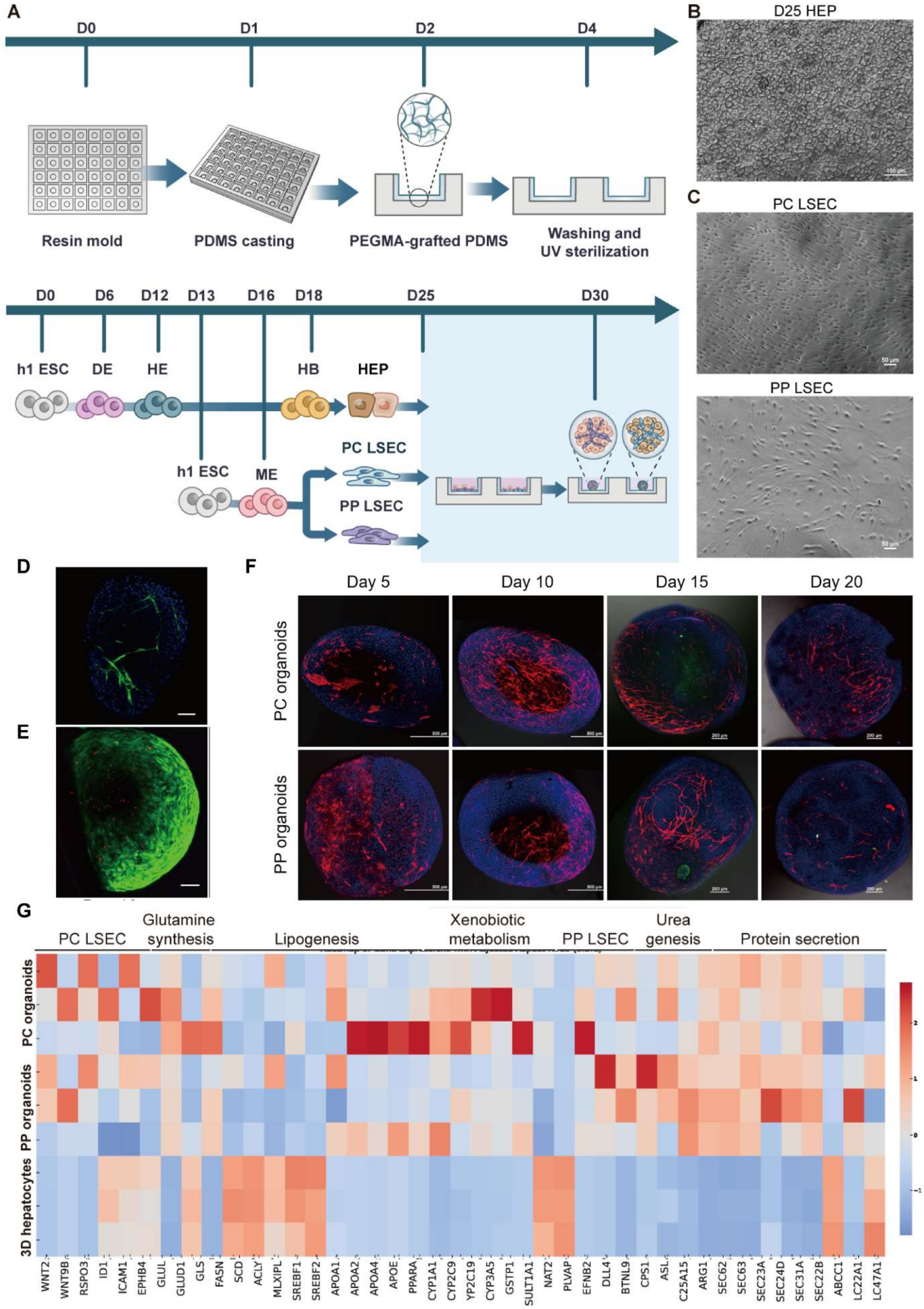

A), The production of the PDMS microwell chip necessitates a four-day process. Differentiation protocols for hepatocytes and liver sinusoidal endothelial cells (LSECs) require 25 and 12 days, respectively. Following the assembly of organoids with day 25 hepatocytes and day 12 LSECs, zonated characteristics are observed after five days of culture. **B),** Bright-field images of hESC-derived day25 hepatocyte, with a scale bar of 100 µm. **C),** Bright-field images of hESC-derived PC LSEC

and PP LSEC, with a scale bar of 50 μm. **D),** Immunofluorescence staining of CD31 in liver organoid z-stack scanning images. The green signal represents CD31, and the blue signal represents DAPI, with scale bar of 100 μm. **E),** Live/Dead staining of the organoids. The green signal represents live cells (Calcein-AM), and the red signal represents dead cells (Propidium Iodide, PI), with a scale bar of 100 μm. **F),** Characterization of liver organoids. Immunofluorescence staining of endothelial cell marker CD31 for liver organoids. The red signal represents CD31, and the blue signal represents DAPI, with scale bar of 500 μm or 200 μm. **G),** RNA sequencing analyzed genes related to *in vivo* peri-central zone function (glutamine synthesis, lipogenesis, xenobiotic metabolism) and *in vivo* peri-portal zone function (urea genesis, protein secretion) of hepatic organoids without LSECs and PC/PP organoids. The heatmap displays the results from three replicate experiments.

Figure S6 The correlation between ID1 and WNT2, related to Figure 4.

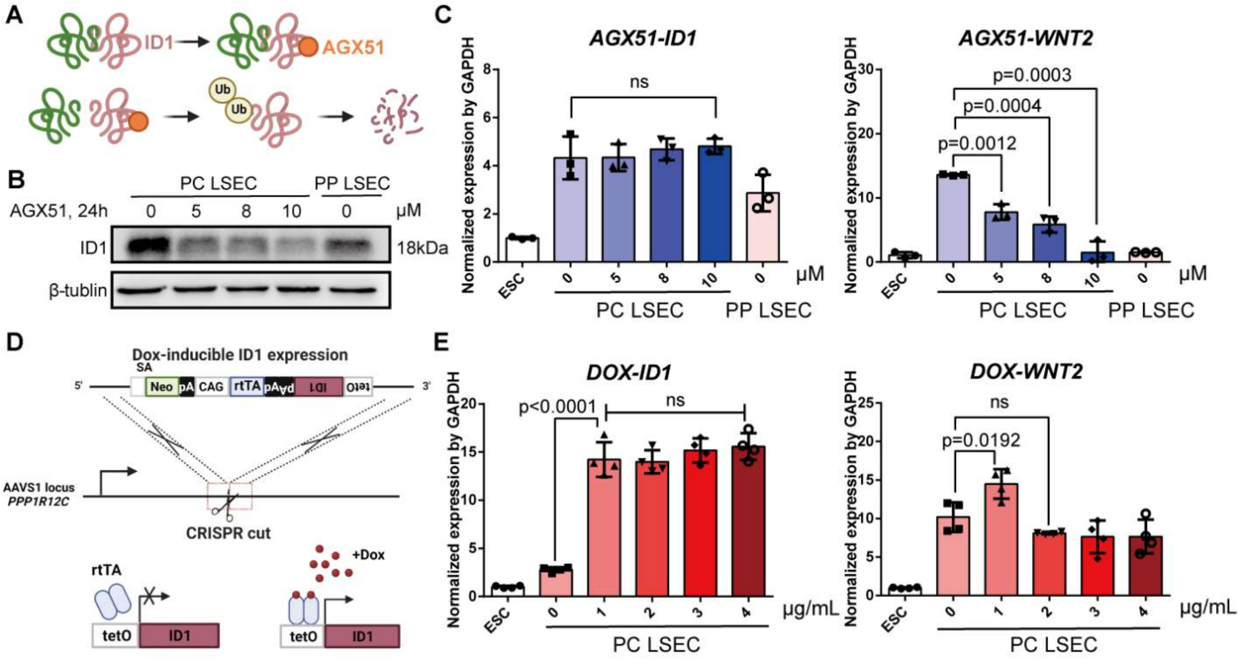

A), The schematic illustrates that AGX51 inhibitors promote the degradation of ID1 by facilitating the ubiquitination of the ID1 protein. **B)**, Western blot analysis for ID1 protein in hESC-derived PC and PP LSEC treated with different doses of AGX51, with β-tublin as a housekeeping protein. **C)**, qPCR results for the expression of *ID1* and *WNT2* genes in hESC-derived PC and PP LSEC treated with different doses of AGX51, with *GAPDH* as a housekeeping gene. Data analysis was performed using one-way ANOVA to compare the differences between various inhibitor doses and the control group (n=3). **D)**, The schematic illustrated the construction of the Dox-inducible ID1-overexpressing Tet-on system in hESC cell line. **E)**, qPCR results for the expression of *ID1* and *WNT2* genes in ID1 KI hESC-derived PC LSEC treated with different doses of DOX, with *GAPDH* as a housekeeping gene. Data analysis was conducted using one-way ANOVA to compare differences between various doses of Dox and the control group (n=4).

Figure S7 Results related to Figure 5.

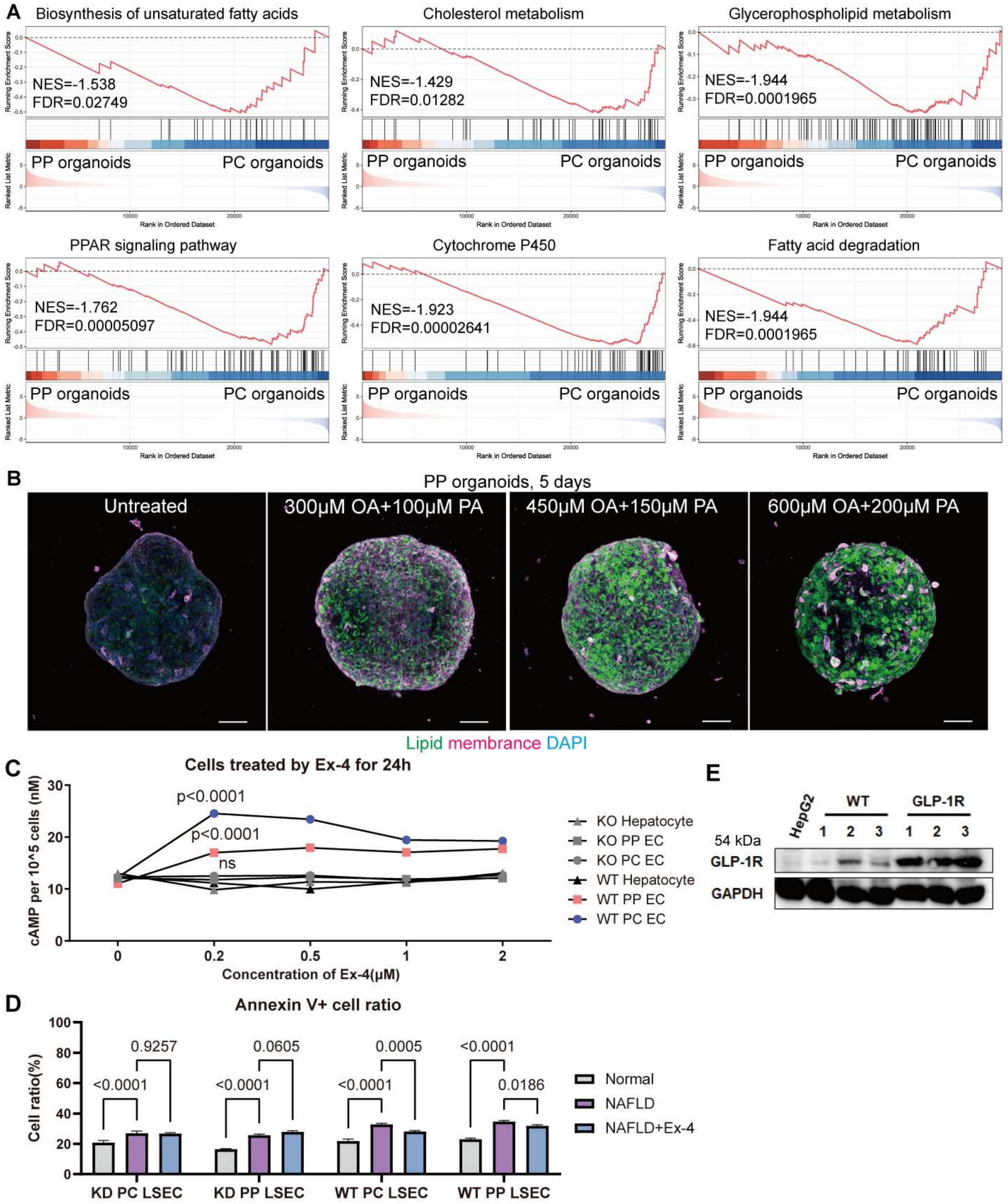

A), Gene Set Enrichment Analysis (GSEA) of MASLD and lipid metabolism pathways in PC and PP Organoids. **B)**, Characterization of lipid accumulation in PP organoids after treatment with different doses of FFAs for 5 days. Cell membrance is visualized in green, Lipid accumulation is visualized in green. The combination of 450 μM OA and 150 μM PA represents the most suitable conditions for inducing MASLD. **C)**, Quantitative analysis of cAMP production in WT hESC-derived PC LSEC, PP LSEC, hepatocyte, and GLP-1R KD hESC-derived PC LSEC, PP LSEC, hepatocyte treated with various concentrations of Ex-4. Data analysis was performed using two-way ANOVA to compare differences in cAMP production across various Ex-4 doses between each sample and WT hep (n=3). **D)**, Flow cytometry quantitative analysis of apoptotic signal Annexin V in WT hESC-derived PC and PP LSEC, and GLP-1R KD hESC-derived PC and PP LSEC treated with Normal, MASLD, MASLD+ 200 nM Ex-4 medium for 24h. Data analysis was performed using two-way ANOVA to compare differences between the Normal and MASLD groups, as well as between the MASLD and MASLD+Ex-4 groups for each type of organoid (n=3). **E)**, Characterization of GLP-1R antibody. HEK293T cells overexpressing GLP1R as a positive control to validate the specificity and accuracy of the GLP-1R band.

Figure S8 **Results related to conclusion.**

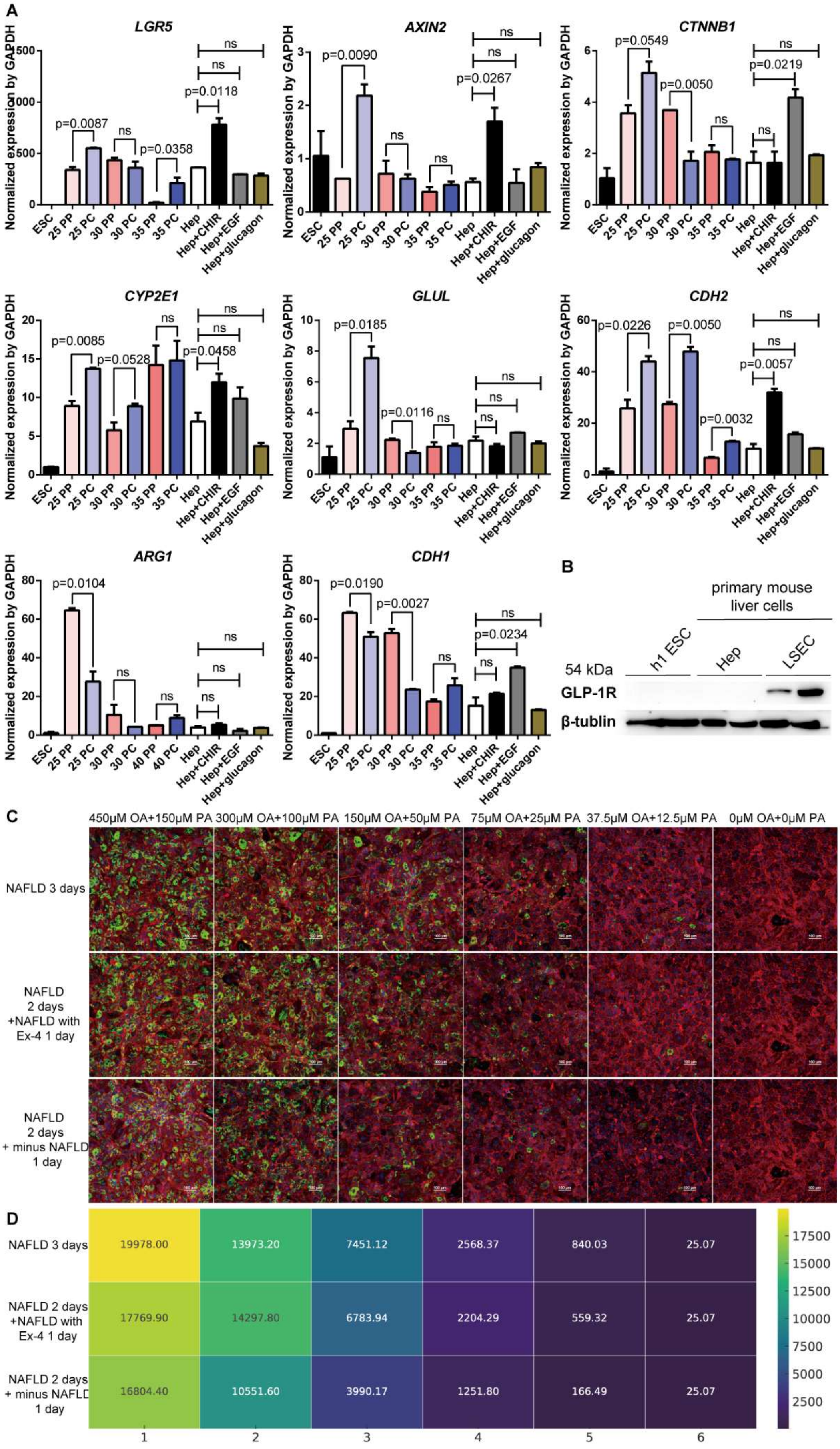

A), qPCR results for the expression of downstream genes of the WNT/β-catenin signaling pathway (*LGR5*, *CTNNB1*, *AXIN2*), primary liver PC zone markers (*GLUL*, *CYP2E1* and *CDH2*) and primary liver PP zone markers (*ARG1* and *CDH1*) in PC and PP organoids, with *GAPDH* as a housekeeping gene. 3D organoid sample include 25PP (day25 hepatocyte co-culture with PP LSEC), 25PC (day25 hepatocyte co-culture with PC LSEC), 30PP (day30 primary mature hepatocyte co-culture with PP LSEC), 30PC (day30 primary mature hepatocyte co-culture with PC LSEC), 35PP (day35 mature hepatocyte co-culture with PP LSEC), 35PC (day35 mature hepatocyte co-culture with PC LSEC). 2D cell sample include hESC, hepatocyte, hepatocyte treated with 2 μM CHIR99021, 20 ng/mL EGF, 5 ng/mL glucagon. Data analysis was performed using one-way ANOVA, separately comparing 3D organoid samples and 2D hepatocyte samples. Groups for comparison were manually selected, and p-values for each group have been indicated. **B),** Western blot analysis for GLP-1R protein in hESC, primary mouse LSEC and hepatocyte, with β-tublin as a housekeeping protein. **C,D)** Characterization **(C)** and quantification **(D)** of lipid accumulation in 2D hepatocyte co-culture with LSEC treated with various concentrations of FFAs and FFAs plus 200 nM Ex-4. Lipid accumulation is visualized in green, while F-actin, characterizing the cytoskeleton, is depicted in red. "Minus MASLD" refers to the use of lower concentrations of FFAs in adjacent conditions.

Table S1 Primers for qPCR.

Gene name	Forward primers	Reverse primers
GAPDH	GGCTGAGAACGGGAAGCTTGTCAT	CAGCCTTCTCCATGGTGGTGAAGA
OCT4	CGACCATCTGCCGCTTTGAG	CCCCCTGTCCCCCATTCCTA
NANOG	GGATGGTCTCGATCTCCTGA	CCTCCCAATCCCAAACAATA
SOX2	CCCCCGGCGGCAATAGCA	TCGGCGCCGGGGAGATACAT
SOX17	CTGCCACTTGAACAGTTTGG	GAGGAAGCTGTTTTGGGACA
FOXA2	CTTCAAGCACCTGCAGATTC	AGACCTGGATTTCACCGTGT
ALB	AATGTTGCCAAGCTGCTGA	CTTCCCTTCATCCCGAAGTT
AAT	ACTGTCAACTTCGGGGACAC	CATGCCTAAACGCTTCATCA
CYP3A4	AGATGCCTTTAGGTCCAATGGG	GCTGGAGATAGCAATGTTCGT
CD32b	GGGATCATTGTGGCTGTG	ATTAGTGGGATTGGCTG
vWF	GCAGTGGAGAACAGTGGTG	GTGGCAGCGGGCAAAC
LYVE1	TGCAGAATTATGGGGATCAC	GGCTGTTTCAACTTGGTCCT
STAB1	ACGCTTCTAACGCCACCTTT	CCACACGATGACGTGGCTAA
STAB2	AGTGGACTATGGACCTAGACCCAAC	AGTAAGCAGCCAAGGCAACAGC
VEGFR-2	GTGACCAACATGGAGTCGTG	CCAGAGATTCCATGCCACTT
VEGFR-3	TGCACGAGGTACATGCCAAC	GCTGCTCAAAGTCTCTCACGA
ALPNR	GCATGGAGGAAGGTGGTGATTT	CAACATGTAGATGGCAGGGATGAG
CXCR4	AGGGAACTGAACATTCCAGAGCGT	AAACGTTCCACGGGAATGGAGAGA
EFNB2	ACGACACTTTGGTTTATGCGGTGC	AAGAGGTCTGCCGTATGTGCTTCA
EPHB4	GTCGTCACCACCAAACTCAA	GGGAACGGGGAGAAAAATTA
HEY2	GAGTGAGAGAGTCGTGTTTC	ACTTCTGTCCCTTTCCTTTC
NR2F2	TGATGTAGCCCATGTGGAAAG	GCTGCCGGACAGTAACATATC
NT5E	CACACGGCATTAGCTGTTATTT	AGGGACAAGTGCAGGTTTAG
WNT2	GATGCGTGCCATTAGCCAG	AGATTCCCGACTACTTCGGAG
WNT9B	TGTGCGGTGACAACCTCAAG	ACAGGAGCCTGATACGCCAT
RSPO3	TGTGCAACATGCTCAGATTACA	TGCTTCATGCCAATTCTTTCCA
ID1	CTGCTCTACGACATGAACGG	GAAGGTCCCTGATGTAGTCGAT
AXIN2	TACACTCCTTATTGGGCGATCA	TTGGCTACTCGTAAAGTTTTGGT
CTNNB1	CATCTACACAGTTTGATGCTGCT	GCAGTTTTGTCAGTTCAGGGA
CYP2E1	GGGAAACAGGGCAATGAGAG	GGAAGGTGGGGTCGAAAGG
ARG1	TGGACAGACTAGGAATTGGCA	CCAGTCCGTCAACATCAAAACT
LGR5	GAGTTACGTCTTGCGGGAAAC	TGGGTACGTGTCTTAGCTGATTA
GLUL	AAGAGTTGCCTGAGTGGAATTTC	AGCTTGTTAGGGTCCTTACGG
CDH1	ATTTTTCCCTCGACACCCGAT	TCCCAGGCGTAGACCAAGA
CDH2	AGCCAACCTTAACTGAGGAGT	GGCAAGTTGATTGGAGGGATG
DLL4	GCCCTTCAATTTCACCTGGC	CAATAACCAGTTCTGACCCACAG
BTNL9	GGACCTGTTCAGTCTGGAAAC	TCTGGACCACCAACTCTTTCT
ANPEP	TTCAACATCACGCTTATCCACC	AGTCGAACTCACTGACAATGAAG
FCN3	TTAATGGTAACCGTACTTTCGCC	TGGTCAGCGTCATAGGTGGTA
ENG	GCATCCTTCGTGGAGCTACC	GAGGAGTGGTCTGGATCGG
ICAM1	ATGCCCAGACATCTGTGTCC	GGGGTCTCTATGCCCAACAA
LXRA	TCTGGAGACATCTCGGAGGTA	GGCCCTGGAGAACTCGAAG
PPARA	ATGGTGGACACGGAAAGCC	CGATGGATTGCGAAATCTCTTGG
FXR	GACTTTGGACCATGAAGACCAG	GCCCAGACGGAAGTTTCTTATT
ACACA	ATGTCTGGCTTGCACCTAGTA	CCCCAAAGCGAGTAACAAATTCT
SREBF1	CGGAACCATCTTGGCAACAGT	CGCTTCTCAATGGCGTTGT
GLP-1R	TGTTCGGGTCATCTGCATCGTG	CTGGAAGGAGGTGAAGGAGAG

Table S2 Key resources table

REAGENT or RESOURCE	SOURCE	IDENTIFIER
Antibodies		
CD34 (FACS)	BD	Cat#555822
CD31 (FACS)	BD	Cat#WM59
FLK1 (FACS)	BD	Cat#560495
CD31 (IF)	abcam	Cat#ab9498
CD32 (IF)	abcam	Cat#ab131051
LYVE1 (IF)	abcam	Cat#ab14917
F8 (IF)	abcam	Cat#ab275376
STAB2 (IF)	abcam	Cat#ab121893
CD13 (IF)	abcam	Cat#ab108310
ICAM1 (IF)	abcam	Cat#ab282575
ALB (IF)	abcam	Cat#ab207327
GS (IF)	abcam	Cat#ab176562
ARG1 (IF)	abcam	Cat# ab96183
β-catenin (IF)	Santa	Cat#sc-7963
E-cadherin (IF)	abcam	Cat#ab40772
N-cadherin (IF)	Invitrogen	Cat#33-3900
ID1 (WB)	abcam	Cat# ab283650
WNT2 (WB)	abcam	Cat#ab109222
GLP-1R (WB)	Invitrogen	Cat#PA597790
Chemicals, peptides, and recombinant proteins		
human Activin A	Peprotech	Cat#120-14E
human BMP4	Peprotech	Cat#120-05
human bFGF	Peprotech	Cat#100-18B
human OSM	Peprotech	Cat#300-10
human EGF	Peprotech	Cat#100-55B
human HGF	Peprotech	Cat#100-39H
human VEGF	Peprotech	Cat#100-20-250
SB431542	Selleck	Cat#S1067
palmitic acid	Sigma	Cat# P0500
oleic acid	Sigma	Cat#364525
Glucagon	Sigma	Cat# G2044
CHIR99021	Sigma	Cat#SML1046
Dex	Sigma	Cat#D4902
L-Ascorbic acid	Sigma	Cat#A8960
Y27632	MedChemExpress	Cat#HY-10583
insulin	Biological Industries	Cat#41-975-100
Doxorubicin	Sigma	Cat# D9891
PEGMA	Sigma	Cat#454990
Exendin-4	MedChemExpress	Cat#HY-13443
LA-BSA	Sigma	Cat#L9530
Transferrin	Sigma	Cat#T1147
Sodium selenite	Sigma	Cat#S5261
Critical commercial assays		
CellTiter-Blue® Cell Viability Assay	Promega	Cat#G8080
Enhanced BCA Protein Assay Kit	Beyotime	Cat#P0010
TRIzol RNA isolation reagents	Invitrogen	Cat#15596018

human IL6 ELISA kit	Elabscience	Cat#E-EL-H6156
Urea Assay Kit	BioAssay Systems	Cat#DIUR-100
Acti-stain 555 phalloidin	Cytoskeleton	Cat#PHDH1-A
Triglyceride detection	Solarbio	Cat#BC0625
Alcohol dehydrogenase activity detection kit	Solarbio	Cat#BC1085
P450-Glo™ CYP3A4 Assay	Promega	Cat#V8901
human CYP2E1 ELISA kit	Ansiang	Cat#AX5554A
Experimental Models: Cell Lines		
hESC	WiCell Institute	H1 ESC
Oligonucleotides		
Primers for quantitative RT-PCR, see Table S1	This paper	N/A
shRNA targeting GLP-1R	CCGGCCACTCACACTTGGAGCTAATCTCGAGATTAGCTCCAAGTGTGAGTGGTTTTT	TRCN0000004705
shRNA targeting GLP-1R	CCGGCCTCATCTTTGTTCGGGTCATCTCGAGATGACCCGAACAAAGATGAGGTTTTT	TRCN0000004706
Amplification of ID1 cDNA (Forward primer)	TCGGTACCGCCACCATGAAAGTCGCCAGTGGCAG	N/A
Amplification of ID1 cDNA (Reverse primer)	TCGTCGACAAGCTTATCAGCGACACAAGATGCGAT	N/A
Software and algorithms		
CFX Manger Software	Bio-Rad	https://www.bio-rad.com/en-kr/sku/1845000-cfx-manager-software?ID=1845000
ImageJ	Version 2.1.0	https://imagej.nih.gov/ij/
Nano Measurer	Version 1.2.0.5	https://nano-measurer.software.informer.com/
FlowJo_V10	BD	https://www.flowjo.com/
GraphPad Prism	Version 7.0	https://www.graphpad.com/scientific-software/prism/
Proteome Discoverer Software	Thermo-Fisher Scientific	Cat#OPTON-30812
Python	Version 3.9.7	https://www.python.org/
RStudio	Version 1.3.1093	https://www.rstudio.com/products/rstudio/
NIS-Elements	version 5.2	https://www.microscope.healthcare.nikon.com/zh_CN/products/software/nis-elements/viewer

Reference

[1] N. Aizarani, A. Saviano, Sagar, L. Mailly, S. Durand, J.S. Herman, P. Pessaux, T.F. Baumert, D. Grun, A human liver cell atlas reveals heterogeneity and epithelial progenitors, Nature, 572 (2019) 199-204.
[2] T.S. Andrews, J. Atif, J.C. Liu, C.T. Perciani, X.Z. Ma, C. Thoeni, M. Slyper, G. Eraslan, A. Segerstolpe, J. Manuel, S. Chung, E. Winter, I. Cirlan, N. Khuu, S. Fischer, O. Rozenblatt-Rosen, A. Regev, I.D. McGilvray, G.D. Bader, S.A. MacParland, Single-Cell, Single-Nucleus, and Spatial RNA Sequencing of the Human Liver Identifies Cholangiocyte and Mesenchymal Heterogeneity, Hepatol Commun, 6 (2022) 821-840.
